# Genome-wide expression profiling of leaves and roots of watermelon in response to low nitrogen

**DOI:** 10.1186/s12864-018-4856-x

**Published:** 2018-06-13

**Authors:** Muhammad Azher Nawaz, Chen Chen, Fareeha Shireen, Zhuhua Zheng, Hamza Sohail, Muhammad Afzal, Muhammad Amjad Ali, Zhilong Bie, Yuan Huang

**Affiliations:** 1 Key Laboratory of Horticultural Plant Biology, Ministry of Education/College of Horticulture and Forestry Sciences, Huazhong Agricultural University, Wuhan, 430070 People’s Republic of China; 2 University College of Agriculture, University of Sargodha, Sargodha, Pakistan; 3 Department of Plant Pathology, and Centre of Agricultural Biochemistry and Biotechnology, University of Agriculture, Faisalabad, Pakistan

**Keywords:** *Citrullus lanatus*, RNA-seq, Nitrogen, Nitrate transporters, Differentially expressed genes, Nitrogen use efficiency

## Abstract

**Background:**

Nitrogen (N) is a key macronutrient required for plant growth and development. In this study, watermelon plants were grown under hydroponic conditions at 0.2 mM N, 4.5 mM N, and 9 mM N for 14 days.

**Results:**

Dry weight and photosynthetic assimilation at low N (0.2 mM) was reduced by 29 and 74% compared with high N (9 mM). The photochemical activity (Fv/Fm) was also reduced from 0.78 at high N to 0.71 at low N. The N concentration in the leaf, stem, and root of watermelon under low N conditions was reduced by 68, 104, and 108%, respectively compared with 9 mM N treatment after 14 days of N treatment. In the leaf tissues of watermelon grown under low N conditions, 9598 genes were differentially expressed, out of which 4533 genes (47.22%) were up-regulated whereas, 5065 genes (52.78%) were down-regulated compared with high N. Similarly in the root tissues, 3956 genes were differentially expressed, out of which 1605 genes were up-regulated (40.57%) and 2351 genes were down-regulated (59.43%), compared with high N. Our results suggest that leaf tissues are more sensitive to N deficiency compared with root tissues. The gene ontology (GO) analysis showed that the availability of N significantly affected 19 biological processes, 8 cell component metabolic pathways, and 3 molecular functions in the leaves; and 13 biological processes, 12 molecular functions, and 5 cell component metabolic pathways in the roots of watermelon. The low affinity nitrate transporters, high affinity nitrate transporters, ammonium transporters, genes related with nitrogen assimilation, and chlorophyll and photosynthesis were expressed differentially in response to low N. Three nitrate transporters (*Cla010066*, *Cla009721*, *Cla012765*) substantially responded to low nitrate supply in the root and leaf tissues. Additionally, a large number of transcription factors (1365) were involved in adaptation to low N availability. The major transcription factor families identified in this study includes MYB, AP2-EREBP, bHLH, C2H2 and NAC.

**Conclusion:**

Candidate genes identified in this study for nitrate uptake and transport can be targeted and utilized for further studies in watermelon breeding and improvement programs to improve N uptake and utilization efficiency.

**Electronic supplementary material:**

The online version of this article (10.1186/s12864-018-4856-x) contains supplementary material, which is available to authorized users.

## Background

Nitrogen (N) is a major component of amino acids, proteins, nucleic acid, chlorophyll and hormones [1]. It is a key macronutrient required for plant growth and development of watermelon. The availability of N affects plant architecture, flowering, fruit development, photosynthesis, and allocation of photosynthates in plants [2–4]. N is absorbed by the roots in the form of nitrate (NO_3_^−^) and ammonium (NH_4_^+^) through the nitrate and ammonium transporters. After uptake these ions are transported to the shoot. During the process of assimilation, NO_3_^−^ is converted to NH_4_^+^ through cytosolic nitrate reductase. Then, NH_4_^+^ is converted to glutamine, glutamate, or glutamate dehydrogenease. These synthesized N compounds serve as precursor for amino acids, proteins, and other N-containing metabolites that are utilized for plant growth and development [4, 5]. Understanding the plant response to nitrogen availability is crucial for sustainable agricultural development [6]. According to a report, under low N conditions plant growth (dry weight) and relative chlorophyll content are substantially reduced compared with optimum N supply [4]. N starvation also reduces leaf area and photosynthetic assimilation capacity leading to reduced plant growth, dry matter accumulation and yield [7, 8].

The absorption of nitrate from the external environment, and transportation and translocation among cells, tissues and organs requires transmembrane proteins. Four protein families are involved in nitrate transport that includes NITRATE TRANSPORTER 1 (NRT1)/PEPTIDE TRANSPORTER (PTR) family (NPF), NITRATE TRANSPORTER 2 (NRT2), CHLORIDE CHANNEL (CLC) family, and SLOWELY ACTIVATING ANION CHANNEL. In *Arabidopsis* and rice 53 and 93 NPF genes has been found. Most of these genes display low nitrate affinities but a few have dual affinities [9]. Most of the NRT2 display high-affinity nitrate transport activity; *Arabidopsis* genome includes 7 NRT2 genes while rice genome has only 4 NRT2 genes. The nitrate transporters play a vital role in nitrate uptake, nitrate signaling, plant growth, lateral root formation, leaf development, stomatal regulation, bud formation, flowering, nitrogen storage, seed development, seed nitrate content, and seed dormancy [9]. The overexpression of nitrate transporters from NRT1 (NPF) and NRT2 families enhances the nitrogen use efficiency of plants. For example, the overexpression of rice OsNRT2.3b enhanced the nitrate and iron uptake and improved the yield under low and high N availability under field condition [10]. N containing fertilizers are applied to all important agronomic and horticultural crops to fulfill their requirement, however, only 30–40% of that fertilizers are utilized by the crops while rest of N is lost through leaching or volatilization and causes environmental pollution along with loss of resources [11]. This situation requires attention, and it becomes important to understand the underlying molecular regulatory mechanism in addition to morphological and physiological plants responses to N deficient conditions. Watermelon is cultivated on a commercial scale across the world, China being the leading producer constitute 66.5% of the world watermelon production. According to National Bureau of Statistics of China, annual use of nitrogenous fertilizer exceeds 24 million tons [12]. A fair share of this nitrogenous fertilizer is utilized for watermelon production.

RNA-Seq is one of the next-generation high throughput sequencing technologies, and it is widely used for transcription profiling, because of low background noise, high sensitivity, reproducibility, dynamic range of expression, and base pair resolution [13]. This technique is utilized to study transcriptomic profiles of different plants under biotic and abiotic stress conditions such as heat and drought stress [14, 15]. Whole transcriptome analyses using RNA-Seq to examine genes involved in N deficiency have been done for *Arabidopsis* [16], maize [17], sorghum [18], cucumber [12, 19], rice [20, 21] and wheat [3]. However, the transcriptome responses of watermelon to low N are not studied yet. Considering the importance of N for plant growth and development, and yield, we executed this study to examine the effect of different levels of N on plant growth; N concentration in different parts of plant, photosynthetic assimilation, and root and leaf transcriptome responses of watermelon seedlings. So far as we know, this is the first study that provides information regarding root and leaf transcriptome responses of watermelon to low N. This study has the potential to reveal novel genes and pathways responsible for adaptation to low N in watermelon. This study can be utilized in crop improvement of watermelon, understanding the mechanism of N metabolism and enhancing the N use efficiency of watermelon plants.

## Results

### Response to growth and physiological traits under low N conditions

#### Plant growth

**Fig. 1:**
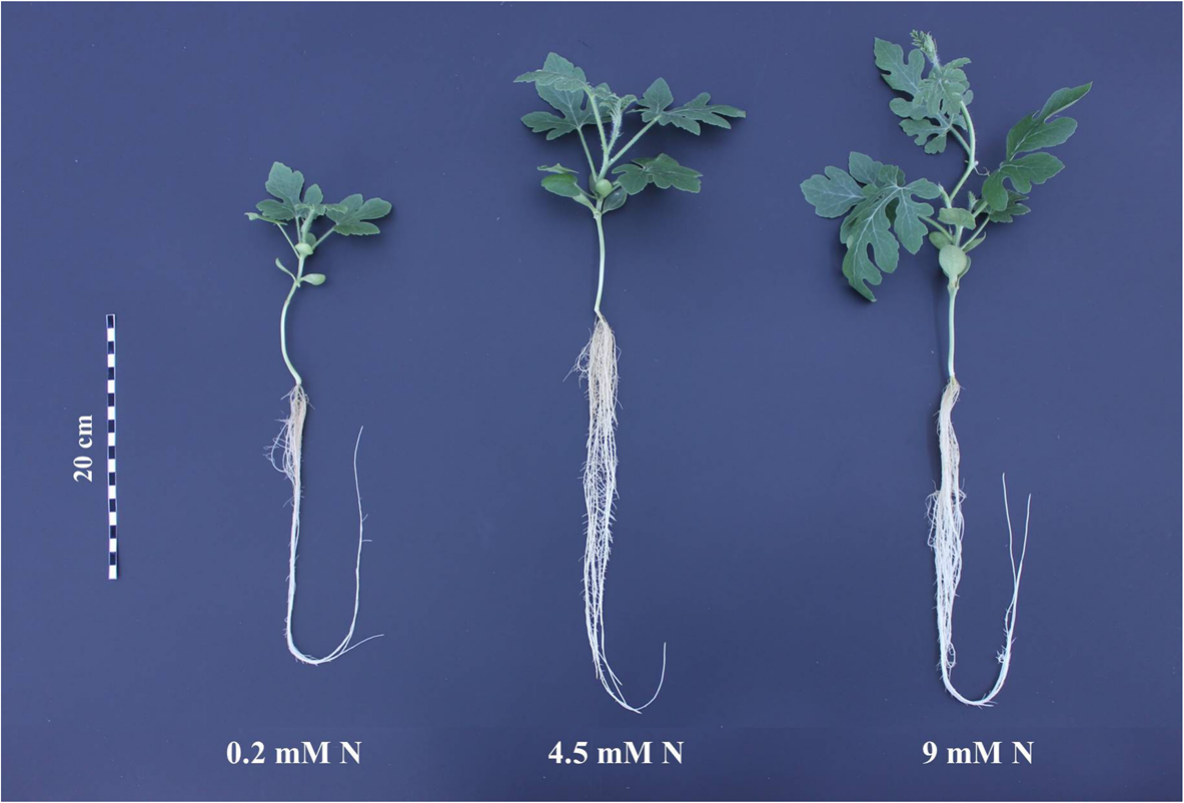
Growth of watermelon plants under different levels of N (0.2 mM, 4.5 mM, 9 mM) grown under hydroponic conditions. Picture was taken after seven days of N treatment

**Fig. 2:**
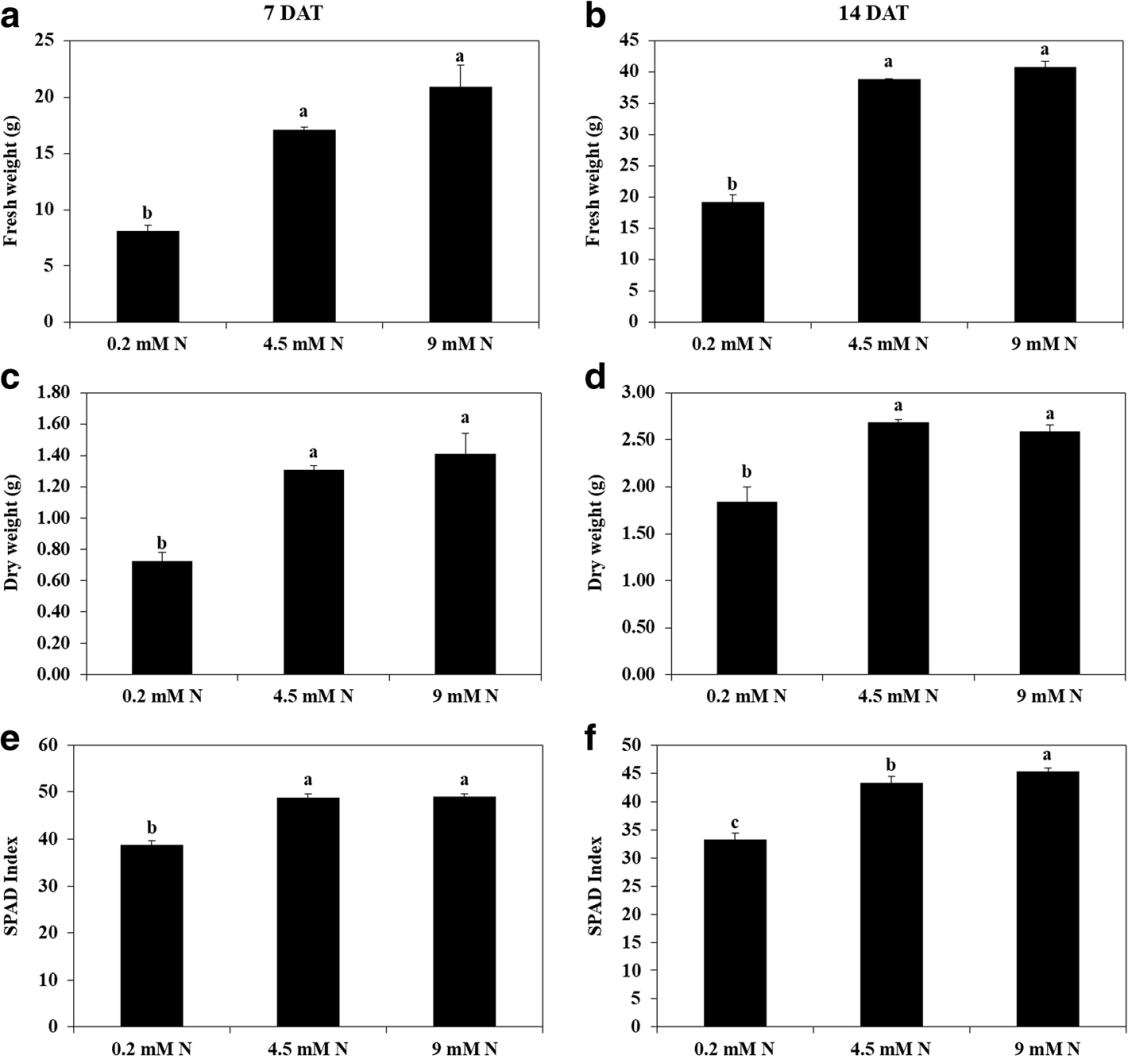
Plant growth (fresh weight and dry weight) and relative chlorophyll measurement (SPAD index) of watermelon seedlings grown under different levels of N (0.2 mM, 4.5 mM, 9 mM). Different letters indicate significant differences by Duncan’s multiple range test at *P* ≤ 0.05. The data for fresh weight (**a**, **b**), dry weight (**c**, **d**) and SPAD index (**e**, **f**) was measured at days 7 and 14 days after N treatment. DAT: days after treatment

The results showed that fresh weight and dry weight of watermelon was significantly affected by different levels of N (Figs. 1, 2a-d). At higher levels of N (4.5 mM and 9 mM) the whole plant fresh weight and dry weight of watermelon plants was 38.77 g/plant and 40.72 g/plant, and 2.68 g/plant and 2.59 g/plant whereas, at low N (0.2 mM) that was only 19.18 g/plant and 1.84 g/plant, respectively at day 14 after N treatment. However, the fresh weight and dry weight of watermelon at 4.5 mM N and 9 mM N was not statistically different from each other (Fig. 2a-d). Relative chlorophyll content (SPAD index) was also altered by different levels of N (Fig. 2e, f). On day 7 after N application, SPAD index was 38.71 at low N, whereas it was increased to 48.92 and 48.96 at 4.5 mM and 9 mM N, respectively. Similar trend was also observed on day 14 after N treatment, SPAD index was only 33.22 at low N whereas it was increased to 43.33 and 45.34 at 4.5 mM and 9 mM N, respectively (Fig. 2e, f).

#### Photosynthetic assimilation

**Fig. 3:**
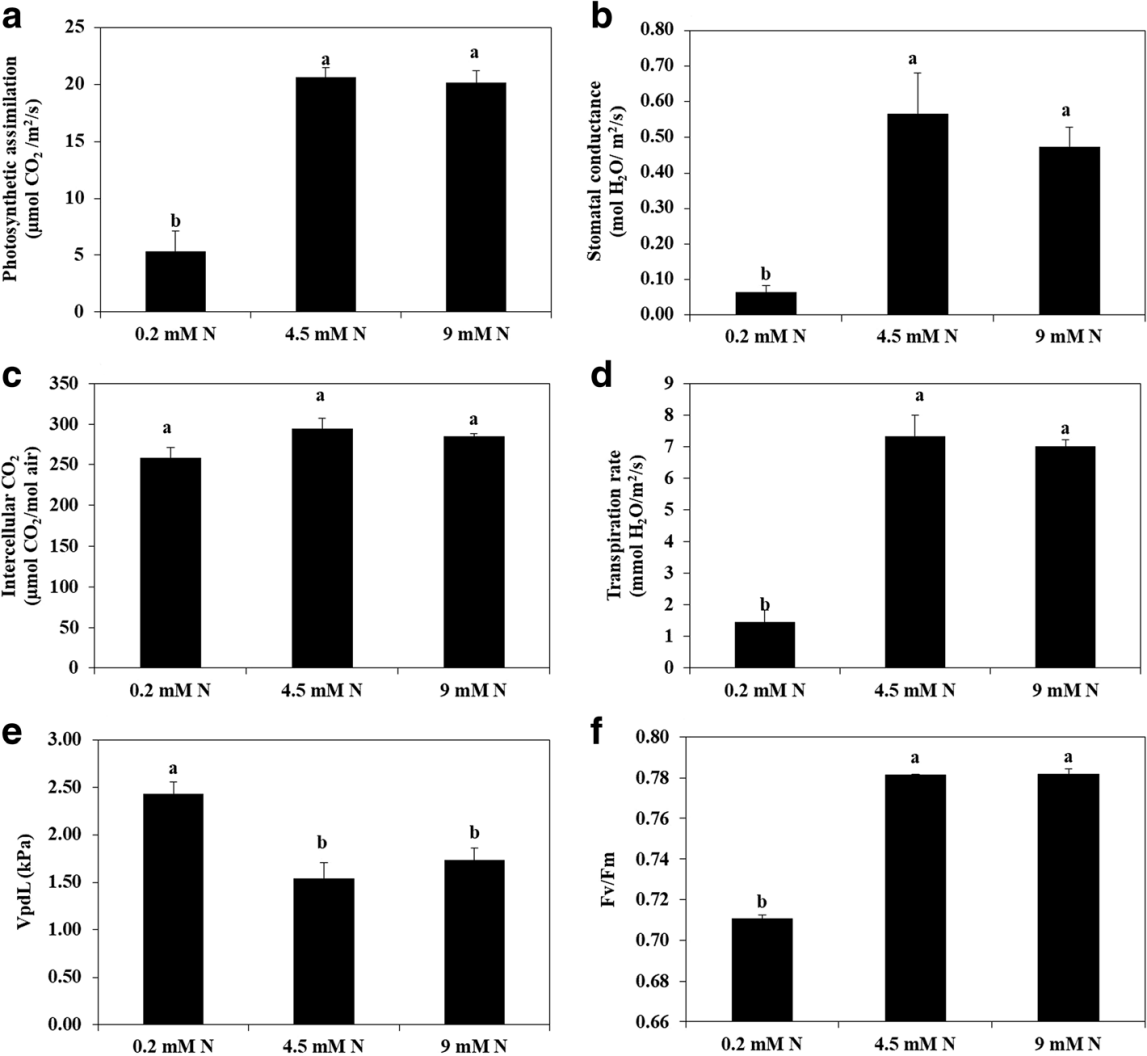
Photosynthetic assimilation (**a**), stomatal conductance (**b**), Intercellular CO_2_ (**c**), transpiration rate (**d**), vapor pressure deficit of leaf (vpdL) (**e**), and maximum photosynthetic efficiency (**f**) of watermelon seedlings grown at different levels of N (0.2 mM, 4.5 mM and 9 mM). Different letters indicate significant differences by Duncan’s multiple range test at *P* ≤ 0.05. The photosynthetic parameters (A-F) were measured at day 14 after N treatment

**Fig. 4:**
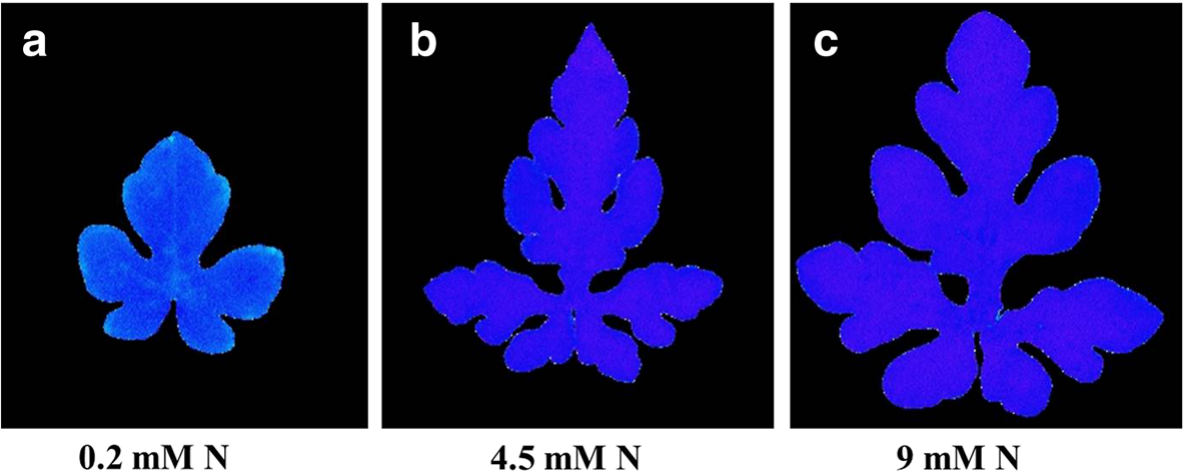
Figures related with maximum photosynthetic efficiency (Fv/Fm) of leaves of watermelon seedlings grown under hydroponic conditions at 0.2 mM N (**a**), 4.5 mM N (**b**), and 9 mM N (**c**). Pictures were taken at day 14 after N treatment

The availability of N to plants affects the chlorophyll content and photosynthetic assimilation of plants. According to the results of this study, rate of photosynthetic assimilation, stomatal conductance, intercellular CO_2_, transpiration rate, vapor pressure deficit, and photosynthetic efficiency of photosystem II (Fv/Fm) was obviously affected by different levels of N (Figs. 3, 4). The photosynthetic assimilation of watermelon leaf grown at low N was reduced by 74% compared with higher levels of N (4.5 mM and 9 MM). Similarly Fv/Fm at low N was 0.71 whereas at higher level of N (4.5 mM and 9 MM) this was improved to 0.78 at day 14 after N treatment (Figs. 3f, 4). All other photosynthetic parameters were also affected by the N availability. Most of the photosynthetic parameters were similar at 4.5 mM N and 9 mM N.

#### Nitrogen concentration in different parts of watermelon

**Fig. 5:**
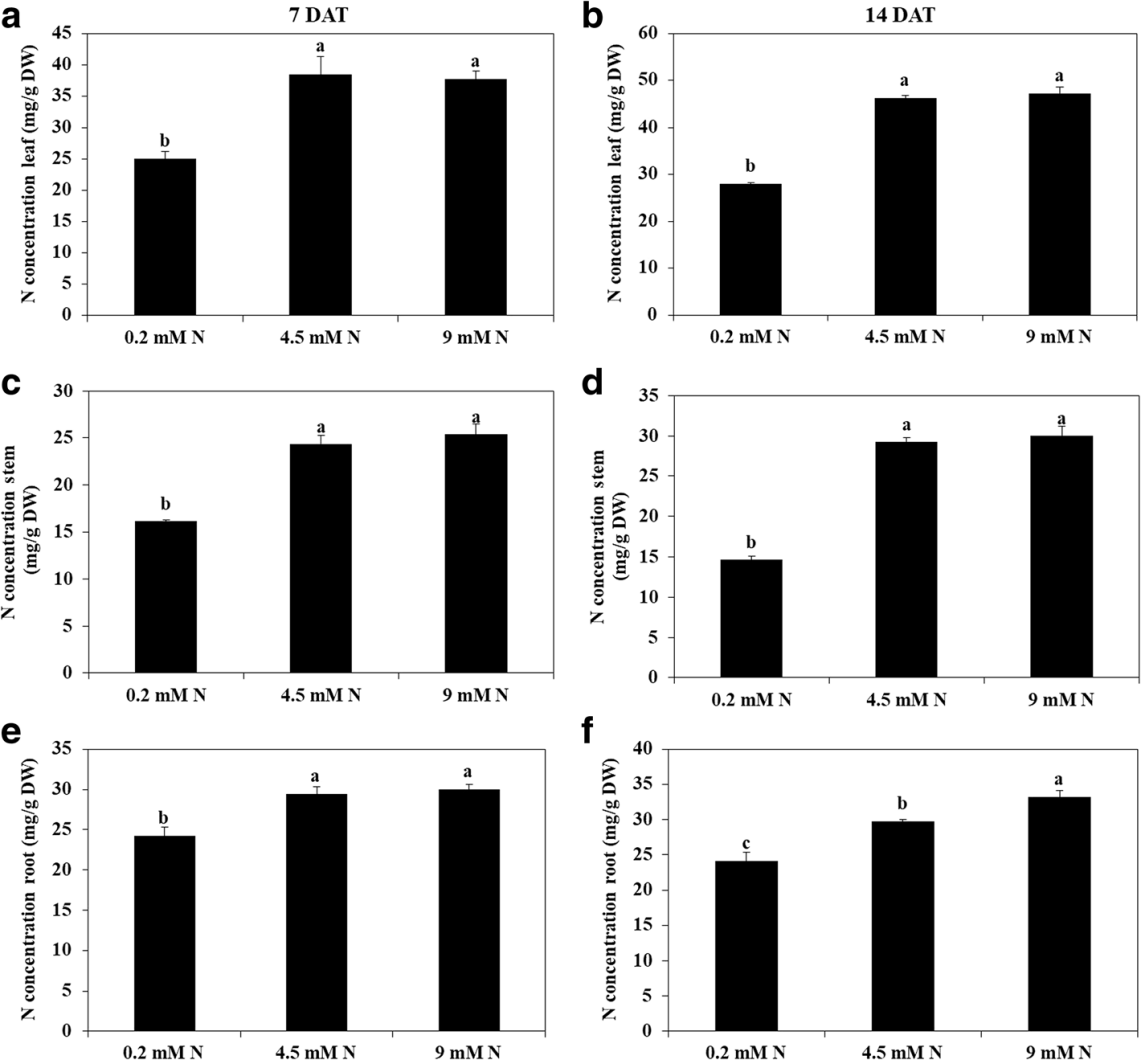
N concentration of leaf (**a**, **b**), stem (**c**, **d**) and root (**e**, **f**) of watermelon seedlings grown under hydroponic conditions at different levels of N (0.2 mM, 4.5 mM and 9 mM). Different letters indicate significant differences by Duncan’s multiple range test at *P* ≤ 0.05. DAT: Days after treatment

The concentration of N in different parts (leaf, stem, and root) of watermelon was affected by the N availability. It was observed that at 4.5 mM N and 9 mM N treatment, the N concentration in leaf, stem, and root was not significantly different from each other; however, at 0.2 mM N treatment, the N concentration was substantially reduced in different parts of plant (Fig. 5). At 0.2 mM N treatment, N concentration in the leaf, stem, and root was reduced by 68, 104, and 108% compared with 9 mM N treatment.

### Transcriptome responses to low N (0.2 mM)

#### Overview of the RNA sequencing data

The transcriptome changes induced by the low N in watermelon leaf and root were investigated by RNA-Seq. A total of 49.97 to 62.81 million reads were generated per sample (Additional file 1: Table S1). Among all the reads, the Q20 and Q30 percentage was more than 96 and 90%, respectively (sequencing error rate was less than 0.02%), and GC content for the libraries was more than 44%. Among all the libraries, the ratio of total mapped reads and multiple mapped reads was 78.37 to 86.76% and 1.07 to 1.61%, respectively, whereas, 77.3 to 85.49% reads were uniquely mapped to the watermelon genome (Additional file 1: Table S2).

#### Analysis of differentially expressed genes (DEGs)

**Fig. 6:**
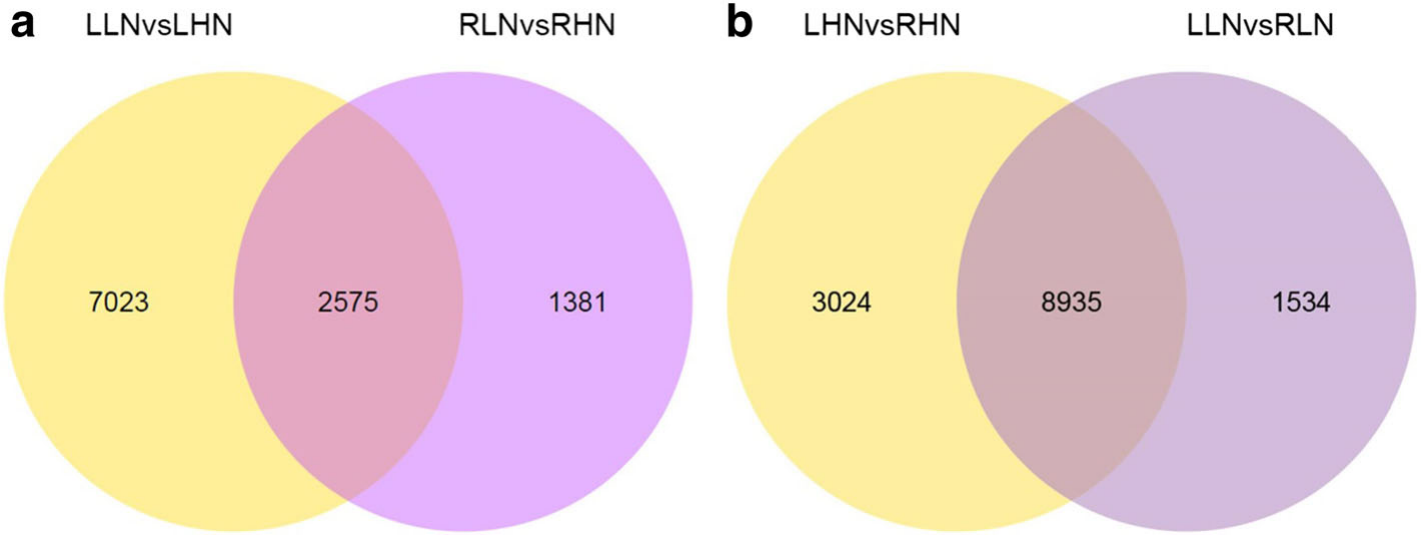
Venn diagrams presenting differential gene expression in the leaf and root of watermelon (**a**, **b**) grown under hydroponic conditions at 0.2 mM and 9 mM N. LHN: leaf high N (9 mM); LLN: leaf low N (0.2 mM); RHN: root high N (9 mM); RLN: roots low N (0.2 mM). The leaf and root samples of watermelon for transcriptome analysis were harvested at day 14 after N treatment

**Fig. 7:**
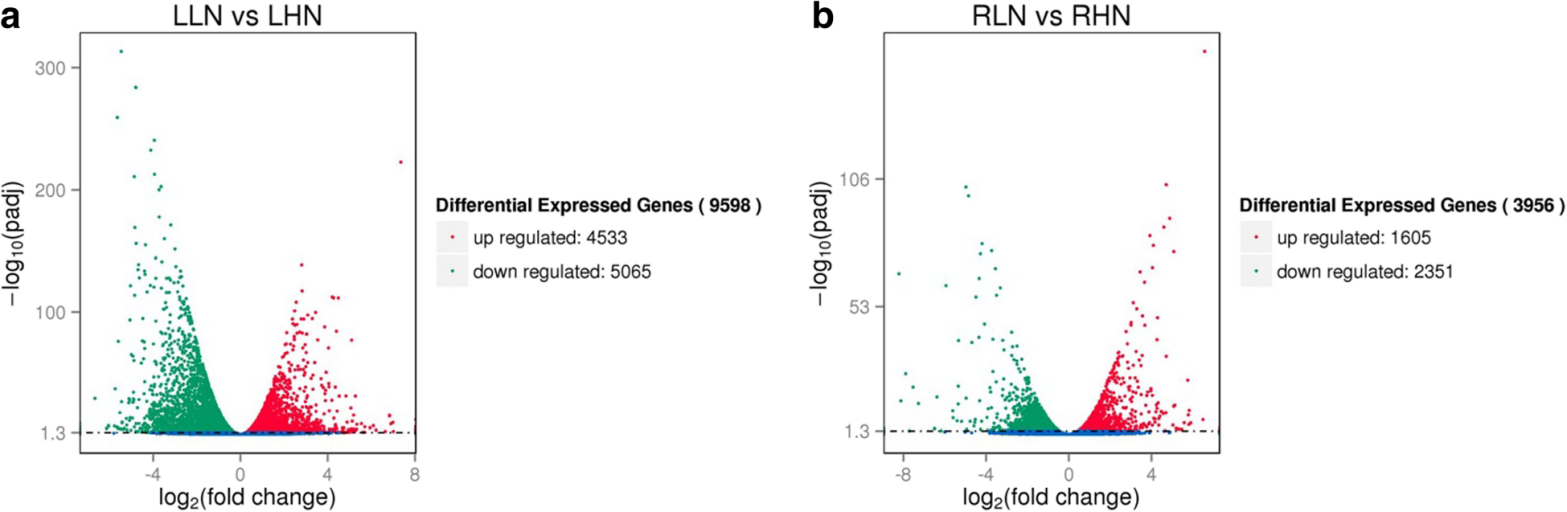
The map representing the number of differentially expressed genes in the leaf (**a**) and root (**b**) of watermelon grown under hydroponic conditions at 0.2 mM and 9 mM N. Red dots represent up-regulated genes and green dots represent down-regulated genes. LHN: leaf high N (9 mM); LLN: leaf low N (0.2 mM); RHN: root high N (9 mM); RLN: roots low N (0.2 mM). The leaf and root samples of watermelon for transcriptome analysis were harvested at day 14 after N treatment

The cluster analysis showed that a large number of genes were differentially expressed in the leaf and root of watermelon grown under low N and high N conditions (Additional file 1: Figure S2; Fig. 6a, b). The differential gene expression analysis of leaves revealed that expression of 9598 genes was significantly (*p* < 0.05) changed when plants were exposed to low N. These included 4533 up-regulated and 5065 genes down-regulated genes (Fig. 7a). The differential gene expression analysis of roots revealed that 3956 transcripts were significantly (*p* < 0.05) altered when plants were exposed to low N. These included 16,054 up-regulated and 2351 down-regulated transcripts (Fig. 7b). These evidences suggest that leaf tissues are more sensitive to N deficiency compared with the root tissues.

**Fig. 8:**
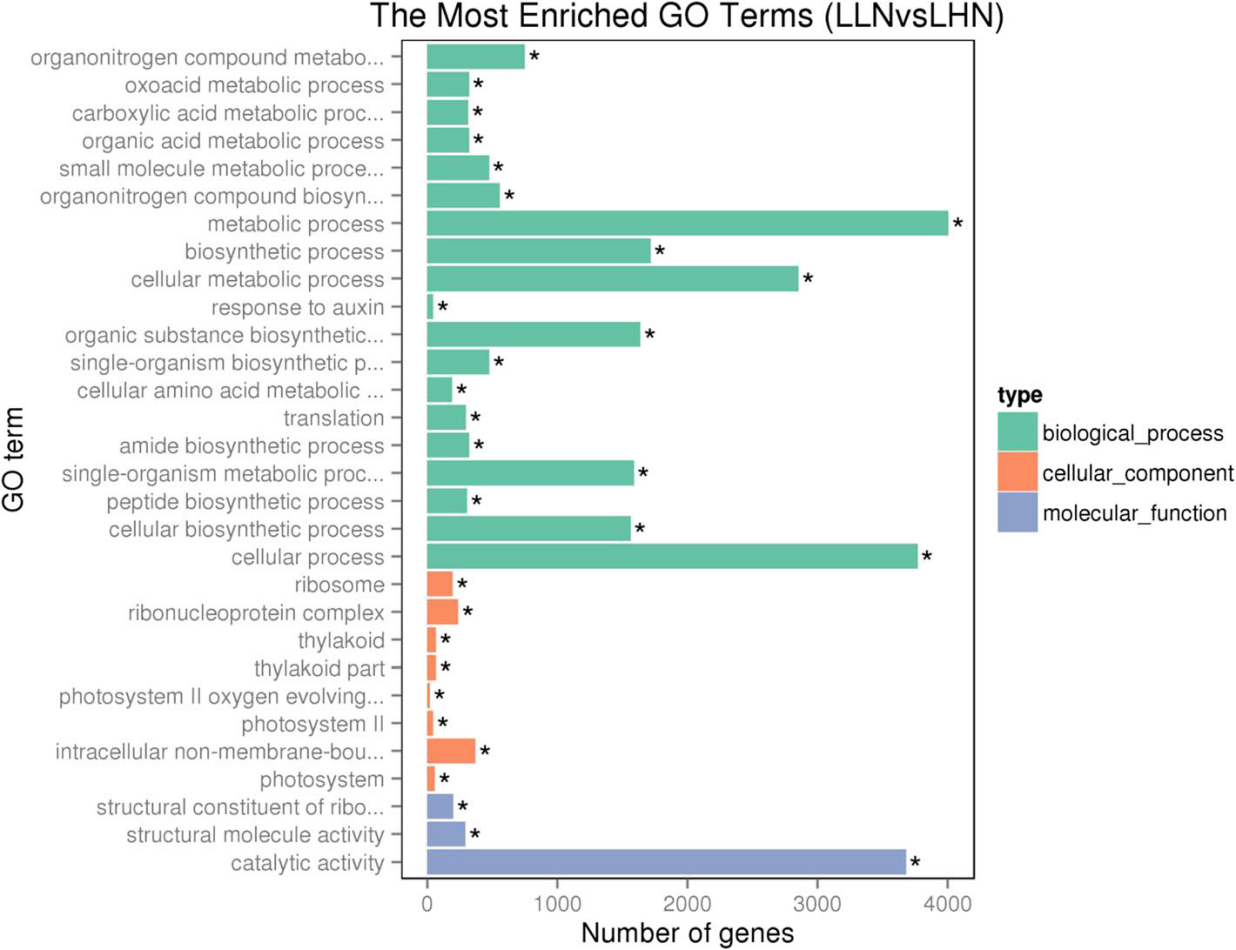
Gene ontology (GO) of differentially expressed genes (DEGs) of the leaf and root of watermelon grown under hydroponic conditions at 0.2 mM N and 9 mM N. LHN: leaf high N (9 mM); LLN: leaf low N (0.2 mM). The leaf samples of watermelon for transcriptome analysis were harvested at day 14 after N treatment

**Fig. 9:**
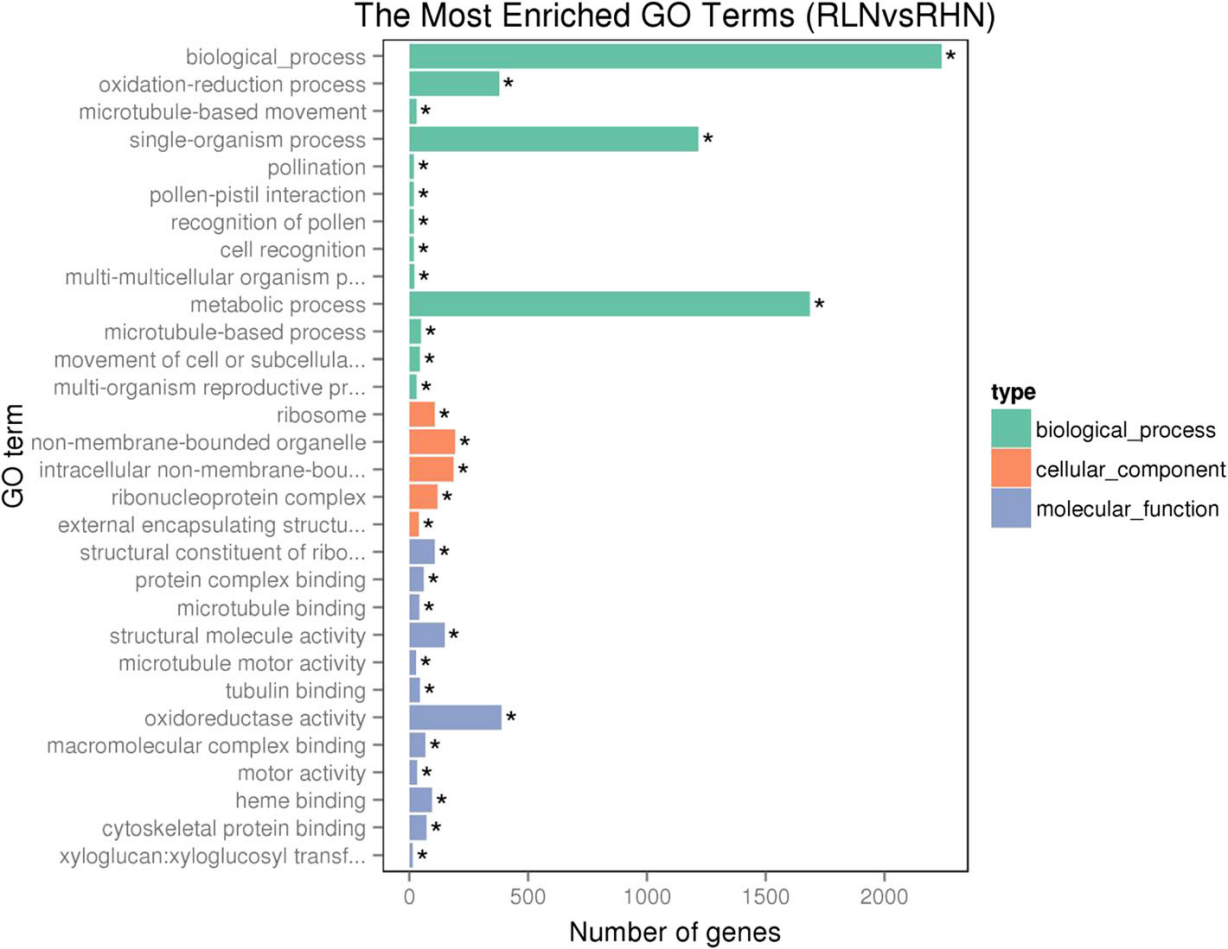
Gene ontology (GO) of differentially expressed genes (DEGs) of the leaf and root of watermelon grown under hydroponic conditions at 0.2 mM and 9 mM N. RHN: root high N (9 mM); RLN: roots low N (0.2 mM). The root samples of watermelon for transcriptome analysis were harvested at day 14 after N treatment

Functional annotation showed that low N significantly (adjusted *p* value < 0.05) affected 19 biological processes, 8 cell component metabolic pathways, and 3 molecular functions in the leaves of watermelon (Fig. 8). Similarly, the availability of N significantly affected 13 biological processes, 12 molecular functions, and 5 cell component metabolic pathways in the roots of watermelon (Fig. 9). The N availability mainly affected the following biological processes in the leaves of watermelon: metabolic process, cellular process, cellular metabolic process, biosynthetic process, organic substance biosynthetic process, single-organism metabolic process, and cellular biosynthetic process; the affected cellular components include intracellular non-membrane-bound organelles, ribonucleoprotein complex, ribosome, thylakoid, and photosystem II; the affected molecular functions include catalytic activity, structural molecule activity, and structural constituents of ribosomes (Fig. 8). For watermelon root, the N availability mainly affected the following biological processes: biological process, metabolic process, single organism process, and oxidation-reduction process; the affected molecular functions include oxidoreductase activity, structural molecule activity, structural constituents of ribosomes, heme binding, and cytoskeletal protein binding; the affected cellular components include non-membrane bounded organelle, intracellular non-membrane-bound organelles, ribonucleoprotein complex, ribosome, and external encapsulating structures (Fig. 9).

**Fig. 10:**
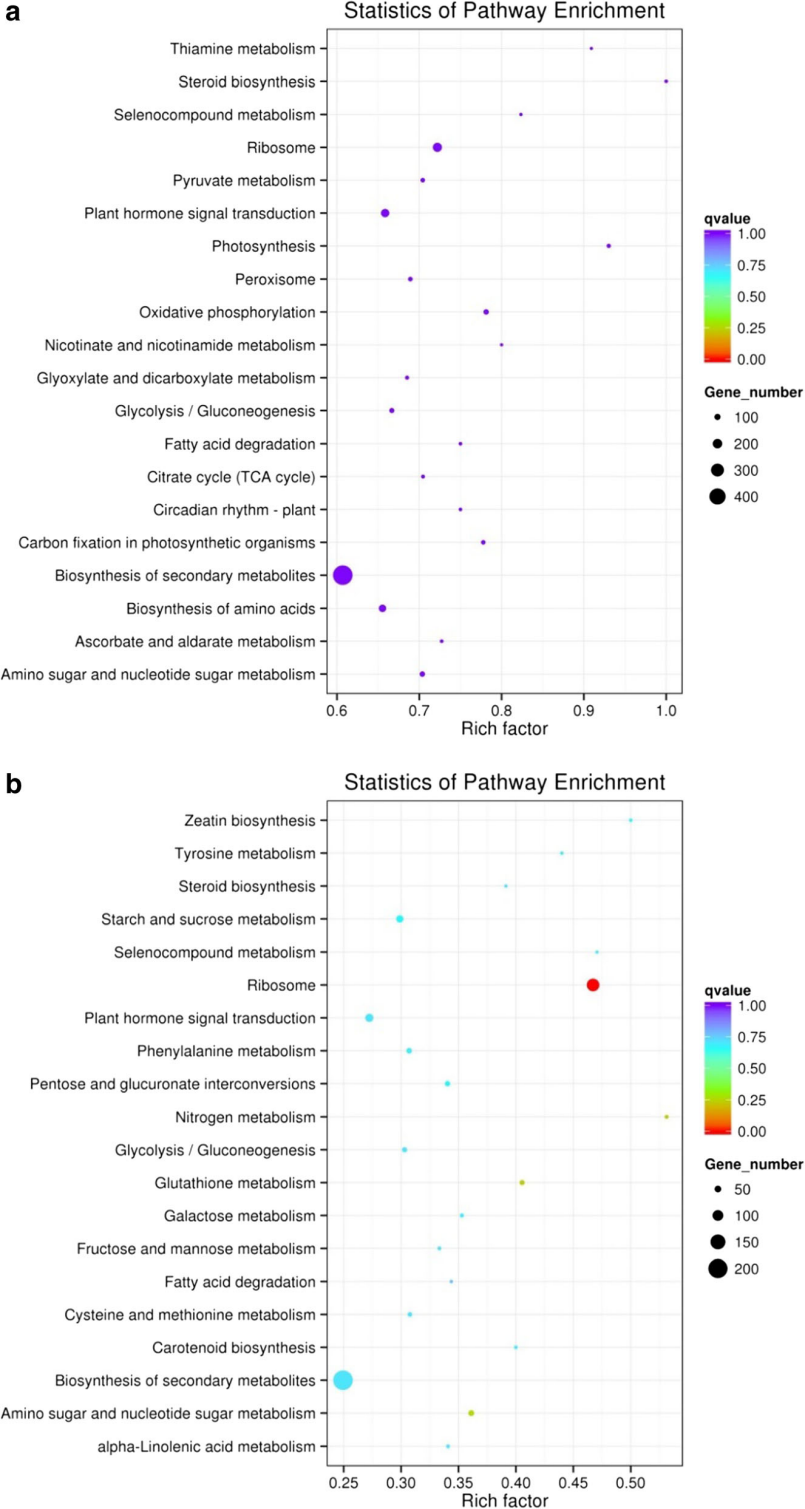
KEEG pathway of differentially expressed genes (GEGs) identified in the leaf (**a**) and the root (**b**) of watermelon grown under hydroponic conditions at 0.2 mM and 9 mM N. The pathway names are provided in the vertical axis, rich factor in the horizontal axis, size of the point represents the number of DEGs, and the color of the dot represents the q value

Kyoto encyclopedia of genes and genomes (KEGG) pathways of differentially expressed genes identified in the leaf and the root of watermelon grown under hydroponic conditions at 0.2 mM N and 9 mM N are represented in Fig. 10. KEGGs analysis showed that maximum genes were differentially expressed for biosynthesis of secondary metabolites, and metabolic pathways. The rich factor represents the ratio between the fraction of pathway genes in the tested set and fraction of pathway genes in the data set. It was observed that the q value was higher for steroid biosynthesis, photosynthesis, thiamin metabolism and selenocompound metabolism pathways in the leaf of watermelon (Fig. 10a). In the leaf tissues, more numbers of genes were affected for biosynthesis of secondary metabolites, ribosome, plant hormone signal transduction and biosynthesis of amino acids (Fig. 10a). In the root tissues, higher q value was observed for nitrogen metabolism, zeatin biosynthesis, selenocompound metabolism, tyrosine metabolism, and ribosome pathways. The pathway for which more number of genes was affected includes biosynthesis of secondary metabolites, ribosome, plant hormone signal transduction and starch and sucrose metabolism pathways (Fig. 10b). The cytoscape presenting protein interaction network analysis of differentially expressed genes of leaf and root of watermelon grown at 0.2 mM and 9 mM N is provided in (Additional file 1: Figure S3).

#### Nitrate transporters gene expression

The nitrate transporters were differentially expressed in the leaves and root of watermelon plants exposed to low N and high N. We observed that in leaf and root tissues, 16 and 11 nitrate transporters were differentially expressed that confirms that leaf tissues are more responsive to N deficiency (Tables 1, 2). In the leaf tissues, the expression of 9 nitrate transporters was increased, and the expression of 7 nitrate transporters was reduced under low nitrate supply (Table 1). Similarly, in the root tissues, the expression of 6 nitrate transporters was increased and expression of 5 nitrate transporters was decreased under low nitrate supply (Table 2). Interestingly, the transcript levels of two high affinity transporters (*Cla005121, Cla005079*) was up-regulated (Table 1) in the leaf tissues, whereas in root tissues the transcript levels of high affinity nitrate transporters (*Cla005121, Cla005080*) was down-regulated under low N compared with high level of N. It was also observed that high affinity transporter *Cla005079* was only expressed in the leaf whereas high affinity transporter *Cla005080* was only expressed in the root (Tables 1 and 2).

**Table 1:** Transcript abundance of nitrate transporters in the leaves of watermelon seedlings grown under hydroponic conditions at low N (LLN) and high N (LHN)

Gene ID	Read count LLN	Read count LHN	Log_2_ fold change	*P* value adjusted	Functional annotation
Cla005079	5.853557617	0	–	0.029828	High-affinity nitrate transporter
Cla005121	175.457244	28.46525568	2.6238	1.38E-13	High affinity nitrate transporter
Cla019130	83.29917455	370.2762809	−2.1522	1.66E-10	Nitrate transporter
Cla015456	456.0266927	1311.044409	−1.5235	3.66E-32	Nitrate transporter
Cla010146	74.32900778	188.8950205	−1.3456	5.36E-10	Nitrate transporter
Cla006969	74.29333102	154.5231455	−1.0565	4.94E-06	Nitrate transporter
Cla012250	941.1639028	1512.747014	−0.68465	6.62E-08	Nitrate transporter
Cla012383	126.5488356	188.1123053	−0.5719	0.008863	Nitrate transporter 1.1
Cla005663	1182.446472	633.7369313	0.89982	3.01E-12	Nitrate transporter
Cla010156	850.8802329	453.1608473	0.90893	2.83E-11	Nitrate transporter
Cla005664	2553.365484	1306.997362	0.96614	2.34E-16	Nitrate transporter
Cla019134	2019.086068	784.9202746	1.3631	4.50E-15	Nitrate transporter
Cla008336	72.35893574	25.24548048	1.5191	1.03E-05	Nitrate transporter
Cla012765	6548.33937	2125.670816	1.6232	7.93E-47	Nitrate transporter
Cla009721	25.8386795	7.748078429	1.7376	0.003778	Nitrate transporter
Cla010066	992.3487772	98.83005253	3.3278	6.67E-49	Nitrate transporter

**Table 2:** Transcript abundance of nitrate transporters in roots of watermelon seedlings grown under hydroponic conditions at low N (RLN) and high N (RHN)

Gene ID	Read count RLN	Read count RHN	Log_2_ fold change	*P* value adjusted	Functional annotation
Cla011567	90.1285445	3631.036515	−5.3323	1.35E-39	Nitrate transporter
Cla005080	253.0667838	1792.908787	−2.8247	0.000232	High-affinity nitrate transporter
Cla005121	2763.368125	11,848.32989	−2.1002	1.83E-06	High affinity nitrate transporter
Cla010146	587.2545171	1483.171479	−1.3366	4.93E-10	Nitrate transporter
Cla019130	83.75683864	160.1580356	−0.93522	0.00595	Nitrate transporter
Cla021894	228.6572853	126.1906883	0.85758	0.021481	Nitrate transporter
Cla010438	439.186976	187.9904145	1.2242	0.003482	Nitrate transporter
Cla012250	179.3884412	51.99757026	1.7866	0.020019	Nitrate transporter
Cla012765	469.2224442	113.2805056	2.0504	2.38E-16	Nitrate transporter
Cla009721	1634.24081	29.11889549	5.8105	0.000105	Nitrate transporter
Cla010066	4932.086137	51.98638611	6.5679	1.09E-159	Nitrate transporter

#### Cytokinin, chlorophyll and photosynthesis gene expression

Cytokinin-related genes were also differentially expressed in the leaf and root tissues of watermelon. Six cytokinin-related DEGs were found in the leaf and 9 cytokinin-related DEGs were found in the root (Tables 3 and 4). The transcript level of cytokinin oxidase/dehydrogenase genes was down-regulated whereas cytokinin riboside 5 & apos-monophosphate phosphoribohydrolase LOG genes was up-regulated under low N (Tables 3 and 4) in the leaf and root of watermelon. The chlorophyll, cytochrome 450, photosystem I, photosystem II, and phytochrome-related genes (171 genes) were also differentially expresses in the leaf of watermelon exposed to low N. Among these genes, the expression of 99 genes was reduced and the expression of 72 genes was increased in the leaf under low N (Additional file 2).

**Table 3:** Transcript abundance of cytokinin-related genes in the leaves of watermelon seedlings grown under hydroponic conditions at low N (LLN) and high N (LHN)

Gene ID	Read count LLN	Read count LHN	Log_2_ fold change	*P* value adjusted	Functional annotation
Cla006831	36.64782854	93.64985562	−1.3535	4.50E-06	Cytokinin oxidase/dehydrogenase
Cla018291	69.00692364	111.3532412	−0.69033	0.015301	Cytokinin riboside 5&apos-monophosphate phosphoribohydrolase LOG3
Cla016833	272.0961406	347.8322316	−0.35428	0.040951	Cytokinin oxidase/dehydrogenase
Cla020868	451.718892	348.4341171	0.37454	0.021043	Cytokinin riboside 5&apos;-monophosphate phosphoribohydrolase LOG
Cla007450	776.2804829	452.9061423	0.77737	2.69E-08	Cytokinin oxidase/dehydrogenase 1
Cla006913	1150.451746	197.031802	2.5457	7.21E-16	Cytokinin riboside 5&apos;-monophosphate phosphoribohydrolase LOG

**Table 4:** Transcript abundance of cytokinin-related genes in the roots of watermelon seedlings grown under hydroponic conditions at low N (RLN) and high N (RHN)

Gene ID	Read count RLN	Read count RHN	Log_2_ fold change	*P* value adjusted	Functional annotation
Cla011204	27.86564563	306.0429613	−3.4572	0.001263	Cytokinin oxidase/dehydrogenase
Cla015247	49.88936013	95.37060822	−0.93481	0.032802	Cytokinin riboside 5&apos-monophosphate phosphoribohydrolase LOG3
Cla020067	200.8449087	326.8136211	−0.70239	0.015792	Cytokinin oxidase/dehydrogenase
Cla007450	1343.027332	2106.949026	−0.64967	0.004406	Cytokinin oxidase/dehydrogenase 1
Cla020828	1272.230628	1983.963586	−0.64103	0.003875	Cytokinin riboside 5&apos-monophosphate phosphoribohydrolase LOG3
Cla022434	2231.485519	1336.985183	0.73902	0.00064	Cytokinin riboside 5&apos-monophosphate phosphoribohydrolase LOG3
Cla002932	190.4533881	110.9073213	0.78008	0.023423	Cytokinin oxidase/dehydrogenase 1
Cla008733	185.3459439	105.5126145	0.81281	0.017292	Cytokinin riboside 5&apos-monophosphate phosphoribohydrolase LOG3
Cla017645	393.9613806	142.9132484	1.4629	1.28E-08	Cytokinin riboside 5&apos-monophosphate phosphoribohydrolase LOG3

Additionally, from the DEGs analysis of leaf we found that 40 genes related with high-affinity nitrate transport (*Cla005079*), potassium transport (*Cla014680*), cytochrome P450 enzymes (*Cla008784*, *Cla020315*, *Cla007079*, and *Cla012616*), MYB transcription factors (*Cla007719*, *Cla013009*, and *Cla011239*), heat stress transcription factor (*Cla016837*) and several unknown proteins (*Cla015045*, *Cla006873*, *Cla019572*, *Cla019353*, *Cla003258*, and *Cla010885*) were only expressed under low N (Additional file 1: Table S3) whereas, 22 genes related with transcriptional regulation (*Cla005997* and *Cla008544*), cytochrome enzymes (*Cla008494* and *Cla007503*), magnesium transport (*Cla011914*) and some unknown proteins (*Cla009881*, *Cla015082*, *Cla014917*, and *Cla001830*) were only expressed under high N (Additional file 1: Table S4). In case of root, six genes (*Cla003430*, *Cla003420*, *Cla015185*, *Cla018599*, *Cla006849* and *Cla012218*) were only expressed under low N and two genes (*Cla016272* and Cla003916) were only expressed under high N. This trend further confirms that low N supply affects more pathways and biochemical process in the leaf tissues compared with the root tissues of watermelon (Additional file 1: Table S5).

#### Identification of low N-responsive transcription factors (TFs)

Considering the importance of TFs to have a major role in regulating stress responsive genes, we analyzed the TFs data obtained from DEGs in the leaf and root. We observed that a total of 1365 transcription factors and regulators belonging to 80 different transcription factor families were expressed (Additional file 3). The major transcription factor families expressed in this study includes MYB (121 genes), AP2-EREBP (117 genes), bHLH (82 genes), C2H2 (72 genes) and NAC (67 genes) (Additional file 3).

### Quantitative real-time PCR for RNA-seq data validation

**Fig. 11:**
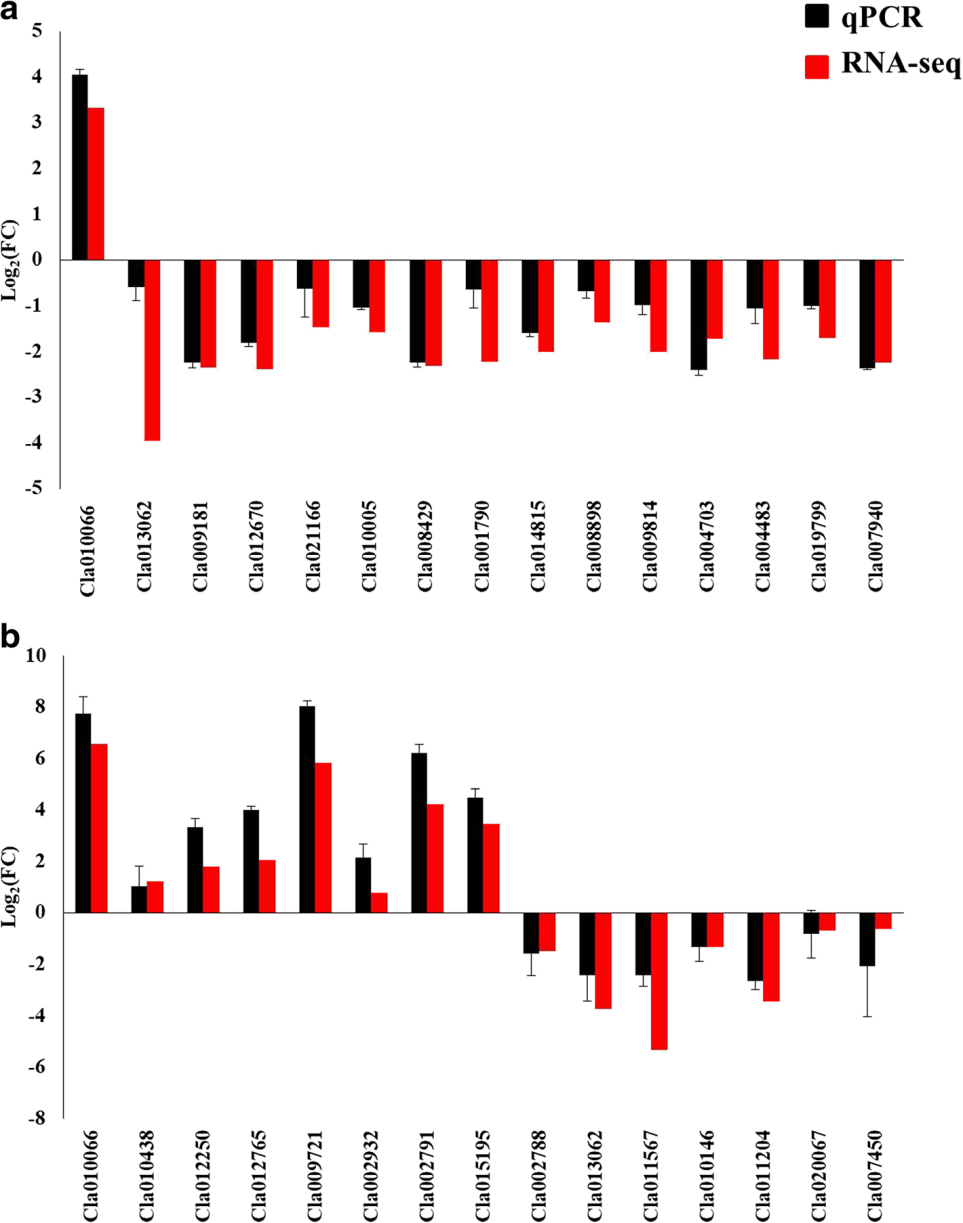
Differential gene expression value of selected genes obtained by total RNA sequencing (RNA-seq) and qPCR in leaf (**a**) and root (**b**) tissues of watermelon seedlings grown under hydroponic conditions exposed to different levels of N (0.2 mM and 9 mM) for 14 days. Bars represent mean log_2_ fold change

Genes related with photosynthesis, nitrate transport and metabolism were selected to carry out quantitative real-time PCR (qRT-PCR) for the validation of RNA-seq data. The results indicated that N deficiency induced changes in the expression of tested genes in the leaf (Fig. 11a) and root (Fig. 11b) of watermelon plants were similar for RNA-seq and qRT-PCR. The correlation coefficient (r) of RNA-seq and qRT-PCR-derived gene expression data was 0.83 and 0.96 for leaf and root, respectively (Additional file 1: Figure S1a, b). Thus the gene expression data obtained from RNA-seq is reliable.

## Discussion

Nitrogen (N) is an important and most abundantly required macronutrient for the plant growth and development. N plays a critical role in a large number of metabolic and biochemical process in plants. N availability directly affects chlorophyll formation and photosynthetic assimilation [4, 5, 22–24]. Low N substantially reduces plant growth. High throughput sequencing technologies such as RNA-Seq is widely used for transcription profiling of different plants growing under biotic and abiotic stresses such as heat and drought stress [13–15]. This technique is also utilized to understand the transcriptomic changes occurred under low N supply in different crops such as rice, sorghum, and cucumber [18–21]. So far as we know, there is no report regarding the transcriptome responses of watermelon to different levels of N supply. Considering this, we performed digital gene expression (DGE) based on illumina sequencing to assess the difference of gene expressions in the leaf and root of watermelon under low and high N supply. Transcriptome data presented in this study provides straight forward information regarding the response of watermelon to low N availability and helps understand the genes and pathways involved.

### DEGs in the leaf

In watermelon leaf under low N, 47.22% of the differentially expressed genes were up-regulated whereas 52.78% genes were down-regulated (Additional file 4). The functional analysis showed that most of the up-regulated genes were related with photosystem, thylakoid, ribosomes, catalytic activity and nitrate transport. Several genes related with nitrate transport and Cytochrome P450 enzymes were up-regulated. This may be a plant strategy to improve the supply of nitrate to leaf tissues and improve photosynthetic efficiency, however, because of limited supply of nitrate (Fig. 4a, b) and reduced leaf relative chlorophyll contents, the photochemical efficiency (Fv/Fm) (Figs. 3f, 4) was not improved leading towards reduced plant growth and development and dry matter accumulation (Figs. 1, 2c, d). This may be attributed to the damaged photosynthetic machinery as a result of photoinhibition and reduced stomatal conductance (Fig. 3b). A potassium transporter (*Cla014680*) was only expressed in the leaf under low nitrate supply (Additional file 1: Table S3) that requires further investigations because potassium has a critical role in plants under stress environment [25, 26]. Thus, this gene (*Cla014680*) might be involved in adaptation to low N.

The down-regulated genes were related with photosystem, photosystem II oxygen evolving complex, thylakoid, ribosomes and ribonucleoprotein complex. For example, the genes related with chlorophyll a and chlorophyll b binding proteins (P4, 3C, 4, 6, 7, 8, 13, 21 and 37) were strongly down-regulated. These proteins are involved in light harvesting complex and serve as conduits for excitation energy to the reaction center of photosystem II [27]. Similarly, several genes related with photosystem I, photosystem II, Cytochrome B561 and Cytochrome P450 enzymes-related protein were down-regulated suggesting the receded photosynthetic efficacy. This reduced photosynthetic assimilation was also evident from the SPAD index (Fig. 2e, f), photosynthetic assimilation (Fig. 3a) and photochemical efficiency data (Fig. 3f).

### DEGs in the root

In watermelon root under low N conditions, 40.57% of the differentially expressed genes were up-regulated whereas 59.43% genes were down-regulated (Additional file 5). The functional analysis showed that most the up-regulated genes were related with nitrate transport and stress pathways. Under limited nitrate supply (< 0.25 mM), high affinity transport system (HATS) is activated [28], however some studies suggest that high affinity transport system also contributes to fulfill nitrate demand of plants at higher levels of nitrate supply (> 0.25 mM) [29, 30]. In our study, similar behavior of some genes involved in HATS (*Cla005121, Cla005080*) was observed; these genes were expressed in the root both under low and high N conditions. A total of three genes involved in HATS were expressed in watermelon, and these genes may be considered for further studies related with nitrate uptake and transport system in watermelon. Some nitrate transporter such as *Cla010066, Cla009721,* and *Cla012765* substantially responded to low N, and their expression was increased by 1.6 to 6.5 fold in the root and leaf tissues of watermelon (Tables 1 and 2). These candidate genes may be focused for future studies to improve the nitrate uptake, transport and utilization efficiency of watermelon. According to a report, overexpression of rice OsNRT2.3b enhanced the nitrate uptake and improved the yield under low and high N availability [10]. Interestingly, under low N condition, ammonium transporters (*Cla007644, Cla007876, Cla014326, Cla018014,* and *Cla021471*) were up-regulated in the root that indicates plants employ alternative strategies to fulfill N requirement. NAC proteins have been extensively reported to play a crucial role in multiple stress tolerance in *Arabidopsis* [31]; herein we also observed the up-regulation of several NAC domain proteins in the watermelon root under low N availability.

The down-regulated genes were related with 40S ribosomal protein, 60S ribosomal protein, ABC transporters, aquaporins, auxin efflux carrier, auxin responsive proteins, auxin transporter-like protein, ethylene responsive transcription factors, gibberellin-regulated proteins, NAC domain proteins, nitrate transporters, zinc finger family proteins, and zinc and phosphorous transporters. Quan et al. [32] also reported that low N induced 1469 differentially expressed genes in the roots of two barley (*Hordeum vulgare*) genotypes. The down-regulated nitrate transporters in the watermelon root includes *Cla011567, Cla010146* and *Cla019130*, additionally, expression of two high affinity nitrate transporters (*Cla005080* and *Cla005121*) was also reduced. Generally under low N availability, the high affinity nitrate transporters are activated but some reports suggest that both high affinity nitrate transporters and low affinity nitrate transporters contributes to fulfill plant nitrate requirement [10, 29, 30].

### Cytokinin and nitrate reductase gene expression

Cytokinin such as zeatine riboside (ZR) and iPA are produced in the root and transported to the shoot where they play an important role in cell division and many other biological process related with growth and development of plants [33, 34]. According to some reports, cytokinins also affect photosynthesis, stomata sensitivity, leaf water content and water use efficiency [35, 36]. Herein, we observed that under low N availability the expression of cytokinin related genes was decreased in the root and leaf tissues leading towards reduced photochemical activity and efficiency, and reduced plant growth (Figs. 2a-d, 3a, f, 4a, c). N availability has a strong influence on N assimilation. Nitrate reductase (NR) is the key enzyme involved in nitrogen assimilation in plants; the activity of this enzyme also affects the nitrate uptake by the roots and nitrate transport to the shoot [4, 37–41]. NR activity is one of the major limitations when N is available in the form of NO_3_^−^ [23, 42]. In this study, the expression of nitrate reductase genes (*Cla023145, Cla002787, Cla002788, Cla013062*) was reduced in the leaf of watermelon under low nitrogen that resulted in disturbance of normal metabolic functions and reduced plant growth and development. In *Chrysanthemum nankingense* under low N availability, a large number of genes involved in N assimilation such as nitrate reductase, nitrite reductase and glutamine synthase were differentially expressed [43]. The reduced relative expression of nitrate reductase genes (*Cla002787, Cla002791, Cla013062, Cla023145*) was also observed in our previous study for the self-grafted watermelon plants under low N [4], whereas the relative expression of these genes was up-regulated in pumpkin-grafted watermelon plants because of enhanced supply of nitrate to watermelon leaves, thus the plant growth and development of watermelon was improved.

### The five main families of transcription factors (TFs) responding to low N

Transcription factors (TFSs) are composed of DNA binding domain that interacts with *cis*-regulator elements of its target genes and a protein to protein interaction domain that facilitates oligmerization between TFs and other regulators [44, 45]. Protein-encoding genes are responsible to regulate the transcription machinery and gene expression. In plants nearly 7% of the gene encodes for TFs, and this ratio is 6% in *Arabidopsis* [46, 47]. N deficiency triggers extensive alterations in the transcriptome of plants. According to a report, under low N supply 48 TFs were up-or down-regulated in soybean [48]. Similarly, 42 TFs were up-or down-regulated in *Arabidopsis* [49]. In our study, a total of 1365 TFs were identified that constitute 5.82% of the total protein coding genes (23,440) predicted in watermelon reference genome (http://cucurbitgenomics.org/organism/1). These 1365 TFs belongs to 80 different transcription factor families. The major transcription factor families identified in this study includes MYB (121), AP2-EREBP (117), bHLH (82), C2H2 (72) and NAC (67) (Additional file 3). These TF families are reported to play a vital role in transcriptional regulation of plants [45].

MYB proteins are key factors in regulatory networks and regulate plant development, metabolism, differentiation and responses to biotic and abiotic stresses [50, 51]. MYB TFs are widely distributed in plants and they are directly involved in regulation of biological process and interact with other TFs [51]. The first MYB TF (C1) was discovered in *Zea mays* [52], after that MYB TFs has been identified in several plants; for example 157 typical R2R3-MYBs encoding genes in maize [53], five 3R-MYB genes and 192 R2R3-MYB genes in *Populus* [54], and 252 MYB genes in soybean [55]. In this study, 121 MYB TF were affected by the availability of N (Additional file 3) that showed MYB TFs are also involved in N metabolism and regulate various biological processes. We observed that 36 MYB TFs were down-regulated and 38 MYB TFs were up-regulated in the leaf tissues of watermelon. Similarly, in root tissues 23 and 19 MYB TFs were down-and up-regulated, respectively (Additional file 6). CmMYB1 is believed to be a critical regulator of N assimilation [56]. Under low N availability, up-regulation of MYB TFs leads towards increased anthocyanin accumulation under high light conditions. The accumulation of anthocyanin helps reduce ROS damage caused by photo-oxidation [49, 57]. AP2-EREBP (ethylene responsive element binding protein) show distinct responses to abiotic stresses such as drought, salinity, low temperature and insect or pathogen attack [58, 59]. AP2-EREBP family proteins are unique to plants and share a highly conserved region of 60–70 amino acids with no obvious similarity outside [60]. About 167-like genes have been identified in maize [61]. The role of AP2-EREBP family TFs has been observed in plants in transcriptional regulation during germination, flowering, carotenogeneis and a target for one phased siRNA. In this study 4 and 9 AP2-EREBP TFs were down-and up-regulated, respectively, in the leaf tissues (Additional file 6).

Basic helix-loop-helix (bHLH) family is also another family of TFs that regulate biological processes such as cell and tissue development, anthocyanin production, light signaling and trichomes development in plants. Heim et al. [62] identified 133 bHLH TFs in *Arabidopsis.* In our study, 82 bHLH TFs were affected by N availability in watermelon leaf and root tissues (Additional file 3). Herein, the expression of 13 bHLH TFs was down-regulated and 14 bHLH TFs was up-regulated in leaf tissues, whereas, 5 bHLH TFs were down-regulated and 4 bHLH TFs were up-regulated in the root tissues of watermelon exposed to low N conditions (Additional file 6). The up-regulation of bHLH TFs helps reduce photo-oxidation damage by increasing the formation of anthocyanin in leaves [49, 57]. C2H2 TFs exist as a superfamily of transcription factors and these are involved in defense responses and many other physiological functions. Ninety-one C2H2 TFs have been found in *Carica papaya* [63]. Zinc finger proteins (ZFPs) belong to C2H2 TFs and these are induced under cold stress and salt stress. The overexpression of ZFP182 in tobacco and rice enhanced the salt stress tolerance [64]. According to another report, the expression of StZFP1 is increased in potato by the infection of late blight pathogen (*Phytophthora infestans*) [65]. In this study, at least 72 member of C2H2 family were affected in the leaf and root tissues of watermelon (Additional file 3) indicating the role of C2H2 TFs towards adaptation to low N availability.

NAC domain proteins are plant-specific transcription factors that play an important role in plant development and regulation of abiotic stress tolerance [31]. NAC domain protein contains highly conserved DNA-binding domain in the N-terminal and diverse transcription activation or repression domain in the C-terminal [66–68]. In *Arabidopsis*, NAC TFs have important role in plant development, senescence and stress regulation. *NAC1* and *AtNAC2* regulate root development [69, 70], CUC1, CUC2, CUC3 controls leaf serration and axillary bud development [71, 72], and SND1 and VND7 trigger the de novo xylem formation and regulate secondary wall synthesis in fibers [73]. In our study, 67 NAC TFs were affected by N availability in watermelon plants (Additional file 3). In the leaf and root tissues of watermelon, 30 and 12; and 14 and 9 NAC TFs were up-and down-regulated respectively, under low N availability (Additional file 6) compared with high N. The coordinated up-regulation of several NAC TFs in the root and leaf tissues exhibits that NAC TFs are involved in adaptation to low N. According to report, ZmDof1-overespression in rice enhances the N and carbon accumulation by increasing N uptake and improving the rate of photosynthesis under low N conditions [74]. In this study, several genes related with Dof family responded under low N availability (Additional file 3) thus, Dof TFs likely have an important role in low N adaptation. Considering the differential expression of transcription factors, it is suggested that the role of TFs in nutrient uptake and assimilation demands attention of plant biologist. This seems a neglected area, thus, requires due considerations to improve the fertilizer use efficiency and nutrient use efficiency of plants. According to the best of our knowledge, this is the first-transcriptome-wide study that provides information for leaf and root transcriptome responses of watermelon plants to low N availability.

## Conclusion

This study provides the physiological and transcriptome responses of leaf and root exposed to low N stress. A large number of DEGs were found in the leaf (9598 genes) and root (3956 genes) exposed to low N. The transcriptome data showed that watermelon leaf is more sensitive to low nitrate supply compared with watermelon root. We identified three nitrate transporters (*Cla010066*, *Cla009721*, *Cla012765*) that substantially responded to low nitrate supply (1.6 to 6.5 fold) in the leaf and root tissues of watermelon. To improve the N uptake and utilization efficiency these genes identified for nitrate uptake and transport can be targeted and utilized in further studies for watermelon breeding and improvement programs. Additionally, a potassium transporter (*Cla014680*) was only expressed in leaf under low N (Additional file 1: Table S3) that requires further investigations to understand the role of potassium transporters to low N adaptation.

## Methods

Watermelon cultivar “Zaojia 8424” (*Citrullus lanatus* (Thunb.) Matsum. and Nakai., Xinjiang Academy of Sciences, China) was utilized as plant material. This study was conducted at the National Center of Vegetable Improvement, Huazhong Agricultural University, China (latitude 30° 27′ N, longitude 114° 20′ E, and altitude 22 m above sea level). The plants were grown under hydroponic conditions in a growth chamber. In this study, plants were grown in full-strength Hoagland solution containing three different levels of N (0.2 mM, 4.5 mM, and 9 mM). The samples for plant growth and N measurement were harvested on days 7 and 14 after N treatment. The photosynthetic assimilation-related parameters were measured on day 14 after N treatment, and the samples for transcriptome analysis were also harvested on day 14 after low N (0.2 mM) treatment. Plant growth and N analysis was performed according to the procedure described in our previous study [4]. The relative chlorophyll content of leaves (third leaf from the top) was measured using SPAD-502 Chlorophyll Meter (Minolta Camera Co., Ltd., Japan). Photosynthetic assimilation, stomatal conductance, intercellular CO_2_ concentration, and transpiration rate of watermelon leaves were measured using a portable photosynthesis system (Li-6400XT, LI-COR, Lincoln, Nebraska, USA). The measuring chamber was controlled to maintain leaf temperature, CO_2_ concentration, and photosynthetic photon-flux density at 25 °C, 360 μM/mol, and 800 μM/m^2^/s, respectively. Chlorophyll fluorescence was measured by utilizing IMAGING-PAM (Heinz Wals GmbH, Germany).

### Transcriptome analysis

#### RNA extraction

The leaf and root samples of watermelon plants growing under 0.2 mM N and 9 mM N were harvested (three replicates) and immediately frozen in the liquid nitrogen. The samples were named as LHN (leaf high N, 9 mM N), and LLN (leaf low N, 0.2 mM N), RHN (root high N, 9 mM N), and RLN (roots low N, 0.2 mM N). Samples were temporary stored at − 80 °C, and further utilized for RNA-Seq. The total RNA of each sample was isolated using the TRIzol® Reagent (Invitrogen) following the manufacturer’s instruction. RNA quality was verified using a 2100 Bioanalyzer (Agilent Technologies, Santa Clara, CA, USA) and checked by RNase free agarose gel electrophoresis. Quality and quantity analysis of total RNA, library construction, and Illumina sequencing were performed by the staff at the Novogene Bioinformatics Technology Co. Ltd., Beijing, China.

#### Library preparation for transcriptome sequencing

A total amount of 3 μg RNA per sample was used as input material for the RNA sample preparations. Sequencing libraries were generated using NEBNext® Ultra™ RNA Library Prep Kit for Illumina® (NEB, USA) following manufacturer’s instructions and index codes were added to attribute sequences to each sample. Briefly, mRNA was purified from total RNA using poly-T oligo-attached magnetic beads. Fragmentation was carried out using divalent cations under elevated temperature in NEBNext First Strand Synthesis Reaction Buffer. First strand cDNA was synthesized using random hexamer primer and M-MuLV reverse transcriptase. Second strand cDNA synthesis was subsequently performed using DNA polymerase I and RNase H. Remaining overhangs were converted into blunt ends via exonuclease/polymerase activities. After adenylation of 3′ ends of DNA fragments, NEBNext Adaptor with hairpin loop structure were ligated to prepare for hybridization. In order to select cDNA fragments of preferentially 150~ 200 bp in length, the library fragments were purified with AMPure XP system (Beckman Coulter, Beverly, USA). Then 3 μl USER Enzyme (NEB, USA) was used with size-selected, adaptor-ligated cDNA at 37 °C for 15 min followed by 5 min at 95 °C before PCR. Then PCR was performed with Phusion High-Fidelity DNA polymerase, Universal PCR primers and Index (X) Primer. At last, PCR products were purified (AMPure XP system) and library quality was assessed on the Agilent Bioanalyzer 2100 system.

#### Clustering and sequencing

The clustering of the index-coded samples was performed on a cBot Cluster Generation System using TruSeq PE Cluster Kit v3-cBot-HS (Illumia) according to the manufacturer’s instructions. After cluster generation, the library preparations were sequenced on an Illumina Hiseq platform and 150 bp paired-end reads were generated.

#### Quality control

Raw data (raw reads) of fastq format were firstly processed through in-house perl scripts. In this step, clean data (clean reads) were obtained by removing reads containing adapter, reads containing ploy-N and low quality reads from raw data. At the same time, Q20, Q30 and GC contents of the clean data were calculated. All the downstream analyses were based on high quality clean data.

#### Reads mapping to the reference genome

Reference genome and gene model annotation files were downloaded from watermelon genome website directly. Index of the reference genome was built using bowtie v2.2.3 and paired-end clean reads were aligned to the reference genome using TopHat v2.0.12. We selected TopHat as the mapping tool because TopHat can generate a database of splice junctions based on the gene model annotation file and thus a better mapping result is obtained compared with non-splice mapping tools.

#### Quantification of gene expression level

HTSeq v0.6.1 was used to count the reads numbers mapped to each gene. Fragments per kiolbase of transcript per million (FPKM) of each gene was calculated based on the length of the gene and reads count mapped to this gene. FPKM considers the effect of sequencing depth and gene length for the reads count at the same time and it is currently the most commonly used method for estimating gene expression [75].

#### Differential gene expression analysis

Differential expression analysis between the treatments (three biological replicates per treatment) was performed using the DESeq R package (1.18.0). DESeq provide statistical routines for determining differential expression in digital gene expression data using a model based on the negative binomial distribution. The resulting *P* values were adjusted using the Benjamini and Hochberg’s approach for controlling the false discovery rate. Genes with an adjusted *P* value < 0.05 (found by DESeq) were considered as differentially expressed genes.

#### GO and KEGG enrichment analysis of differentially expressed genes

Gene ontology (GO) enrichment analysis of differentially expressed genes was done by the GOseq R package, in which gene length bias was corrected. GO terms with corrected *P* value < 0.05 were considered significantly enriched by differentially expressed genes. KEGG is a database for understanding high-level functions and utilities of the biological system such as the cell, organism and ecosystem from molecular-level information, particularly large-scale datasets generated by genome sequencing and other high-through put experimental technologies. We used KOBAS software to test the statistical enrichment of differentially expressed genes in KEGG pathways.

### Validation of RNA-seq gene expression using reverse transcriptase real time PCR (qRT-PCR)

Thirty genes were selected for the validation of RNA-Seq data of leaf and root. cDNA was synthesized from the total RNA of three independent biological replicates of leaf and root tissues exposed to 0.2 mM N and 9 mM N. The sequences of the selected genes (Additional file 1: Table S6) were taken from the watermelon reference genome (http://cucurbitgenomics.org/search/genome/1). The specific primers were designed as described by Kong et al. [76] using the Primer 3 software. All the qRT-PCR reactions were arranged on a 384-well plate. qRT-PCR was performed using LightCycler480 SYBR Green 1 Master Kit (Roche diagnostics, Mannheim, Germany) according to the described procedure. PCR amplification was done using Quantstdio™ 7 Flex Real-Time PCR system, Life technologies™, Carlsbad, CA, USA that includes 3 min of pre-incubation at 95 °C, followed by a 40 cycles of 95 °C for 10 s, 58 °C for 15 s, and 72 °C for 15 s. The PCR product was quantified with the same kit described above, and the data was analyzed using 2^-∆∆Ct^ method [77]. *Actin* (*Cla007792*) was utilized as a reference gene.

## Additional files

Additional file 1: **Table S1.** Summary of sequencing data quality of leaves and roots of watermelon grown under hydroponic conditions at low N (0.2 mM) and high N (9 mM); **Table S2.** Summary of total, multiple and uniquely mapped reads of leaves and roots of watermelon grown under hydroponic conditions at low N (0.2 mM) and high N (9 mM); **Table S3.** Transcript abundance of the genes that were only expressed under low N (LLN) in the leaves of watermelon seedlings grown under hydroponic conditions; **Table S4.** Transcript abundance of the genes that were only expressed under high N (LHN) in the leaves of watermelon seedlings grown under hydroponic conditions; **Table S5.** Transcript abundance of the genes that were expressed either under low N (RLN) or high N (RHN) in the roots of watermelon seedlings grown under hydroponic conditions; **Table S6.** The list of primer sequences used for qRT-PCR analysis; **Table S7.** *Arabidopsis thaliana* Ortholog genes to the selected candidate genes that substantially responded to low N (0.2 mM) compared with high N (9 mM) in the leaf and root of watermelon; **Figure S1.** Correlation between expression value of selected genes obtained by RNA-seq and qPCR in the leaf (a) and root (b) tissues of watermelon seedlings grown under hydroponic conditions exposed to different levels of N (0.2 mM and 9 mM) for 14 days. FC: fold change; r: correlation coefficient; **Figure S2.** Hierarchical cluster analysis map presenting differential gene expression in the leaf and root of watermelon grown under hydroponic conditions at 0.2 mM and 9 mM N. LHN: leaf high N (9 mM); LLN: leaf low N (0.2 mM); RHN: root high N (9 mM); RLN: roots low N (0.2 mM). Samples for transcriptome analysis were harvested after 14 days of N treatment; and **Figure S3.** The cytoscape presenting protein interaction network analysis of differentially expressed genes of leaf and root of watermelon grown under hydroponic conditions at 0.2 mM and 9 mM N. (DOCX 1230 kb)

Additional file 2: The expression of chlorophyll, cytochrome 450, photosystem I, II, and phytochrome-related genes in watermelon leaves exposed to different levels of nitrogen supply (0.2 mM, 9 mM) (XLSX 27 kb)

Additional file 3: Transcription factors (TFs) found to be affected in the leaf and root tissues of watermelon exposed to different levels of nitrogen (0.2 mM, 9 mM) (XLSX 76 kb)

Additional file 4: The expression pattern of different genes in the leaf tissues of watermelon exposed to different levels of nitrogen (0.2 mM, 9 mM) (XLSX 1109 kb)

Additional file 5: The expression pattern of different genes in the root tissues of watermelon exposed to different levels of nitrogen (0.2 mM, 9 mM). (XLSX 453 kb)

Additional file 6: The summary of five major transcription factor families in leaf and root tissues of watermelon exposed to different levels of N (0.2 mM, 9 mM) (XLSX 38 kb)

## Abbreviations

DEGs: Differentially expressed genes
Fv/Fm: Photochemical activity
GO: Gene ontology
HATS: High affinity transport system
iPA: Isopentyl adenosine
KEGG: Kyoto encyclopedia of genes and genomes
LHN: Leaf high nitrogen
LLN: Leaf low nitrogen
N: Nitrogen
NH_4_^+^: Ammonium
NO_3_^−^: Nitrate
PCR: Polymerase chain reaction
RHN: Root high nitrogen
RLN: Root low nitrogen
RNA-seq: RNA sequencing
TFs: Transcription factors
ZR: Zeatine riboside
