# Supplementary material for: Genome-wide expression profiling of leaves and roots of watermelon in response to low nitrogen
Source: BMC Genomics. 2018 Jun 13;19:456. doi: 10.1186/s12864-018-4856-x (PMC6001020; doi:10.1186/s12864-018-4856-x)
Supplement: Supplementary file 1 — Table S1. Summary of sequencing data quality of leaves and roots of watermelon grown under hydroponic conditions at low N (0.2 mM) and high N (9 mM); Table S2. Summary of total, multiple and uniquely mapped reads of leaves and roots of watermelon grown under hydroponic conditions at low N (0.2 mM) and high N (9 mM); Table S3. Transcript abundance of the genes that were only expressed under low N (LLN) in the leaves of watermelon seedlings grown under hydroponic conditions; Table S4. Transcript abundance of the genes that were only expressed under high N (LHN) in the leaves of watermelon seedlings grown under hydroponic conditions; Table S5. Transcript abundance of the genes that were expressed either under low N (RLN) or high N (RHN) in the roots of watermelon seedlings grown under hydroponic conditions; Table S6. The list of primer sequences used for qRT-PCR analysis; Table S7. Arabidopsis thaliana Ortholog genes to the selected candidate genes that substantially responded to low N (0.2 mM) compared with high N (9 mM) in the leaf and root of watermelon; Figure S1. Correlation between expression value of selected genes obtained by RNA-seq and qPCR in the leaf (a) and root (b) tissues of watermelon seedlings grown under hydroponic conditions exposed to different levels of N (0.2 mM and 9 mM) for 14 days. FC: fold change; r: correlation coefficient; Figure S2. Hierarchical cluster analysis map presenting differential gene expression in the leaf and root of watermelon grown under hydroponic conditions at 0.2 mM and 9 mM N. LHN: leaf high N (9 mM); LLN: leaf low N (0.2 mM); RHN: root high N (9 mM); RLN: roots low N (0.2 mM). Samples for transcriptome analysis were harvested after 14 days of N treatment; and Figure S3. The cytoscape presenting protein interaction network analysis of differentially expressed genes of leaf and root of watermelon grown under hydroponic conditions at 0.2 mM and 9 mM N. (DOCX 1230 kb) [file 12864_2018_4856_MOESM1_ESM.docx]

**Supplementary Data**

**Table S1** Summary of sequencing data quality of leaves and roots of watermelon grown under hydroponic conditions at low N (0.2 mM) and high N (9 mM)

| Sample Name | Raw Reads | Clean Reads | Clean Bases | Error Rate (%) | Q20 (%) | Q30 (%) | GC Content (%) |
| --- | --- | --- | --- | --- | --- | --- | --- |
| LHNR_1_ | 55111656 | 53903538 | 8.08G | 0.02 | 96.09 | 90.32 | 45.73 |
| LHNR_2_ | 62813224 | 61419236 | 9.2G | 0.02 | 96.18 | 90.54 | 45.72 |
| LHNR_3_ | 53917012 | 52728822 | 7.9G | 0.02 | 96.18 | 90.52 | 45.66 |
| LLNR_1_ | 54310404 | 53200424 | 7.97G | 0.02 | 96.24 | 90.64 | 44.47 |
| LLNR_2_ | 59311278 | 56431934 | 8.46G | 0.02 | 96.85 | 92.20 | 44.72 |
| LLNR_3_ | 49975874 | 47395636 | 7.11G | 0.02 | 96.42 | 91.31 | 44.60 |
| RHNR_1_ | 54920800 | 52162090 | 7.82G | 0.02 | 96.49 | 91.54 | 44.15 |
| RHNR_2_ | 56268884 | 53547634 | 8.03G | 0.02 | 96.80 | 92.20 | 44.29 |
| RHNR_3_ | 53338612 | 50674034 | 7.6G | 0.02 | 96.05 | 90.60 | 44.11 |
| RLNR_1_ | 54086048 | 51309478 | 7.7G | 0.02 | 96.37 | 91.35 | 44.00 |
| RLNR_2_ | 51459214 | 48916154 | 7.34G | 0.02 | 96.68 | 91.97 | 43.98 |
| RLNR_3_ | 58009818 | 55147480 | 8.27G | 0.02 | 96.59 | 91.76 | 44.06 |

LHN: leaf high N (9mM); LLN: leaf low N (0.2 mM); RHN: root high N (9mM); RLN: roots low N (0.2 mM). Samples were harvested after 14 days of N treatment

**Table S2** Summary of total, multiple and uniquely mapped reads of leaves and roots of watermelon grown under hydroponic conditions at low N (0.2 mM) and high N (9 mM)

| Sample Name | Total Reads | Total Mapped | Multiple Mapped | Uniquely Mapped |
| --- | --- | --- | --- | --- |
| LHNR_1_ | 53903538 | 46192453 (85.69%) | 769931 (1.43%) | 45422522 (84.27%) |
| LHNR_2_ | 61419236 | 52382217 (85.29%) | 987549 (1.61%) | 51394668 (83.68%) |
| LHNR_3_ | 52728822 | 45619957 (86.52%) | 844592 (1.6%) | 44775365 (84.92%) |
| LLNR_1_ | 53200424 | 45546193 (85.61%) | 688633 (1.29%) | 44857560 (84.32%) |
| LLNR_2_ | 56431934 | 48962360 (86.76%) | 718990 (1.27%) | 48243370 (85.49%) |
| LLNR_3_ | 47395636 | 40585874 (85.63%) | 595547 (1.26%) | 39990327 (84.38%) |
| RHNR_1_ | 52162090 | 43935949 (84.23%) | 735355 (1.41%) | 43200594 (82.82%) |
| RHNR_2_ | 53547634 | 45177653 (84.37%) | 679624 (1.27%) | 44498029 (83.1%) |
| RHNR_3_ | 50674034 | 42056974 (83%) | 655813 (1.29%) | 41401161 (81.7%) |
| RLNR_1_ | 51309478 | 41958885 (81.78%) | 570155 (1.11%) | 41388730 (80.66%) |
| RLNR_2_ | 48916154 | 38337076 (78.37%) | 524108 (1.07%) | 37812968 (77.3%) |
| RLNR_3_ | 55147480 | 44933932 (81.48%) | 747344 (1.36%) | 44186588 (80.12%) |

LHN: leaf high N (9mM); LLN: leaf low N (0.2 mM); RHN: root high N (9mM); RLN: roots low N (0.2 mM). Samples were harvested after 14 days of N treatment

**Table S3** Transcript abundance of the genes that were only expressed under low N (LLN) in the leaves of watermelon seedlings grown under hydroponic conditions

| Sr. No. | Gene ID | Read count LLN | Read count LHN | *P* value adjusted | Functional annotation |
| --- | --- | --- | --- | --- | --- |
|  | Cla015937 | 36.43847843 | 0 | 0.003414 | Organ-specific protein S2 |
|  | Cla001080 | 33.45870202 | 0 | 7.64E-13 | Polygalacturonase |
|  | Cla015045 | 33.12281564 | 0 | 0.00266 | Unknown Protein |
|  | Cla014680 | 30.61622468 | 0 | 1.51E-12 | Potassium transporter |
|  | Cla000569 | 24.28117718 | 0 | 0.007886 | Major latex protein |
|  | Cla006873 | 22.45194823 | 0 | 4.61E-07 | Unknown Protein |
|  | Cla019572 | 17.06764385 | 0 | 0.001671 | Unknown Protein |
|  | Cla016837 | 15.66358382 | 0 | 1.69E-06 | Heat stress transcription factor A3 |
|  | Cla019731 | 14.07651026 | 0 | 7.11E-06 | FAD-binding domain-containing protein |
|  | Cla017272 | 13.91077881 | 0 | 0.002256 | Polygalacturonase |
|  | Cla008784 | 13.07539539 | 0 | 0.005153 | Cytochrome P450 71D11 (Fragment) |
|  | Cla020315 | 11.98916397 | 0 | 0.015303 | Cytochrome P450 |
|  | Cla009271 | 11.51732364 | 0 | 0.01576 | Major allergen Mal d 1.0502 |
|  | Cla002274 | 11.47944795 | 0 | 7.15E-05 | CASP-like protein RCOM_1174750 |
|  | Cla015783 | 11.2560754 | 0 | 0.000127 | Lysine/histidine transporter |
|  | Cla020152 | 10.65513733 | 0 | 0.00016 | GDSL esterase/lipase |
|  | Cla007079 | 9.870047505 | 0 | 0.0003 | Cytochrome P450 |
|  | Cla018151 | 9.447828221 | 0 | 0.000416 | Glutathione S-transferase |
|  | Cla000417 | 9.055464579 | 0 | 0.007968 | Metallothionein-II protein |
|  | Cla012029 | 9.023410006 | 0 | 0.000674 | Serine/threonine-protein kinase DDB_G0283821 |
|  | Cla013036 | 8.489168173 | 0 | 0.012128 | Legumin 11S-globulin |
|  | Cla004669 | 8.327343304 | 0 | 0.041161 | Cysteine/Histidine-rich C1 domain family protein |
|  | Cla003380 | 7.966852966 | 0 | 0.009229 | Peroxidase |
|  | Cla005187 | 7.775353724 | 0 | 0.002062 | Calmodulin binding protein-like protein (Fragment) |
|  | Cla019353 | 7.704932174 | 0 | 0.045063 | Unknown Protein |
|  | Cla009174 | 6.668994366 | 0 | 0.005115 | CASP-like protein POPTRDRAFT_798217 |
|  | Cla003258 | 6.286177151 | 0 | 0.037409 | Unknown Protein |
|  | Cla006989 | 6.286177151 | 0 | 0.037409 | AT-hook DNA-binding protein (Fragment) |
|  | Cla016188 | 6.065857359 | 0 | 0.011314 | AT-hook DNA-binding protein (Fragment) |
|  | Cla005079 | 5.853557617 | 0 | 0.029828 | High-affinity nitrate transporter |
|  | Cla007719 | 5.714913448 | 0 | 0.013689 | MYB transcription factor |
|  | Cla013009 | 5.54268833 | 0 | 0.014674 | MYB transcription factor |
|  | Cla010885 | 5.372843411 | 0 | 0.018204 | Unknown Protein |
|  | Cla004815 | 5.270367288 | 0 | 0.022677 | Beta-glucosidase G3 |
|  | Cla022576 | 5.270367288 | 0 | 0.022677 | Glutamate receptor |
|  | Cla011239 | 4.699103585 | 0 | 0.032156 | R2R3-MYB transcription factor-like protein |
|  | Cla021346 | 4.638228463 | 0 | 0.032926 | Heavy-metal-associated domain--containing protein |
|  | Cla012616 | 4.627828213 | 0 | 0.033056 | Cytochrome p450 |
|  | Cla009952 | 4.416382294 | 0 | 0.041687 | Serine carboxypeptidase-like 13 |
|  | Cla011631 | 4.407508421 | 0 | 0.041827 | Amino acid permease |

Samples for transcriptome analysis were harvested after 14 days of N treatment

**Table S4** Transcript abundance of the genes that were only expressed under high N (LHN) in the leaves of watermelon seedlings grown under hydroponic conditions

| Sr. No. | Gene ID | Read count LLN | Read count LHN | *P* value adjusted | Functional annotation |
| --- | --- | --- | --- | --- | --- |
|  | Cla006755 | 0 | 22.10304176 | 1.12E-09 | UDP-glycosyltransferase 74 F1 |
|  | Cla004671 | 0 | 18.02759036 | 1.45E-07 | B-cell receptor-associated protein 31-like protein |
|  | Cla022438 | 0 | 13.1455952 | 7.84E-06 | Glyoxal oxidase |
|  | Cla007503 | 0 | 11.24788538 | 4.57E-05 | Cytochrome b561 |
|  | Cla009881 | 0 | 11.21104566 | 5.70E-05 | Unknown Protein |
|  | Cla011370 | 0 | 9.969428382 | 0.00017 | Trehalase |
|  | Cla019416 | 0 | 9.889894555 | 0.000175 | Cysteine-rich repeat secretory protein 55 |
|  | Cla011784 | 0 | 8.445950825 | 0.000689 | Expansin S2 |
|  | Cla008544 | 0 | 8.22273445 | 0.000922 | MYB transcription factor |
|  | Cla020146 | 0 | 8.157671467 | 0.000944 | Patatin-like protein 3 |
|  | Cla008494 | 0 | 7.699879492 | 0.001375 | Cytochrome P450 |
|  | Cla006133 | 0 | 7.315202261 | 0.001949 | ABC transporter G family member 8 |
|  | Cla015082 | 0 | 7.060650996 | 0.002636 | Unknown Protein |
|  | Cla003829 | 0 | 6.943363196 | 0.002753 | Pectinesterase |
|  | Cla005325 | 0 | 6.074499241 | 0.007014 | GDSL esterase/lipase |
|  | Cla013377 | 0 | 5.269784008 | 0.014139 | Acetyltransferase GNAT family protein expressed |
|  | Cla005997 | 0 | 4.981504649 | 0.019204 | Transcriptional regulator superman |
|  | Cla011914 | 0 | 4.641000474 | 0.026625 | Magnesium transporter nipa2 |
|  | Cla014917 | 0 | 4.438674026 | 0.035151 | Unknown Protein |
|  | Cla001830 | 0 | 4.241852399 | 0.037584 | Unknown Protein |
|  | Cla010065 | 0 | 4.214543389 | 0.03794 | Invertase/pectin methylesterase inhibitor family protein |
|  | Cla010795 | 0 | 4.166344453 | 0.038587 | Glutamine dumper 2 |

Samples for transcriptome analysis were harvested after 14 days of N treatment

**Table S5** Transcript abundance of the genes that were expressed either under low N (RLN) or high N (RHN) in the roots of watermelon seedlings grown under hydroponic conditions

| Sr. No. | Gene ID | Read count RLN | Read count RHN | | *P* value adjusted | Functional annotation |
| --- | --- | --- | --- | --- | --- | --- |
|  | Cla003430 | 14.12435988 | 0 | | 0.012904 | ECA1 protein |
|  | Cla003420 | 11.80865051 | 0 | | 0.001132 | Unknown Protein |
|  | Cla015185 | 7.863606604 | 0 | | 0.017942 | Legumin 11S-globulin |
|  | Cla018599 | 7.177384129 | 0 | 0.024879 | | Expansin |
|  | Cla006849 | 7.159992278 | 0 | 0.042259 | | UDP glycosyltransferase |
|  | Cla012218 | 6.430201455 | 0 | 0.048565 | | Unknown Protein |
|  | Cla016272 | 0 | 23.14769059 | 0.003326 | | Unknown Protein |
|  | Cla003916 | 0 | 8.381556932 | 0.013727 | | Fasciclin-like arabinogalactan protein |

Samples for transcriptome analysis were harvested after 14 days of N treatment

**Table S6** The list of primer sequences used for qRT-PCR analysis

| Gene accession number | Forward primer(5’-3’) | Reverse primer(5’-3’) |
| --- | --- | --- |
| *Cla010066* | GAATCATGATGGGTTCCGCC | GACGAATGTAGCGAGGGAGA |
| *Cla013062* | TCCTCTCTCTCAGCTCCCTT | ACCCATCTCTCTCCTCCACT |
| *Cla009181* | TTCTCTTCAGGCCTTCTCCG | CTCAACCGTGGCTTTCAGAG |
| *Cla012670* | CTGGTGCTGTGGTTGCTAAG | CCTTGTGAATGGAGCAGCAA |
| *Cla021166* | CACCCTCTTCTCCTCTTGCA | GCTCGAAGTACCGCTCCTTA |
| *Cla010005* | TCAAAGCCCACCAGACTCAT | TTGAACAGAACCCAAGCCAC |
| *Cla008429* | TGTTCTGGCTTGCAACTACG | TCCTTCCTTCGTTCTTGGCT |
| *Cla001790* | TGGACGAGATTGAAGGACCC | CCAAGAAAGATGAGCCACGG |
| *Cla014815* | CAAACAACTTGGGGATGGGG | AGCCAGACTCTTCATCCTCT |
| *Cla008898* | GTTCCGGTGTTCTTCCATGG | GTGTTCAGAAGGCATCGTCC |
| *Cla009814* | GCTCCATCCGCAAACAGAAA | TTAAGGCCAAGAACGACACC |
| *Cla004703* | TGACGTTGATGGAAAAGCATACT | TCAACAACTGTGCGCAATTT |
| *Cla004483* | AGGGAGAATGTGAGCAAGCA | GCCAATAGAACCCCAAGCAA |
| *Cla019799* | GGGAGGGGTAAAGTGTAGGG | AGCCAGAATCCACCCAAGAA |
| *Cla007940* | CGCTTCATCCAACACCAGAG | GTTCTGTAGGCTGGGAGGTT |
| *Cla010438* | AAGCTGGGAAGGTACCTCAC | CAGACCCAAATCCCGAAACG |
| *Cla012250* | AGGAAACGATGGAGAAGGCT | CAGCCAAGTTGCCATTGACT |
| *Cla012765* | TAAGCTCCCTGTCACATGCA | ATATCTCTCGAATGCCCCGG |
| *Cla009721* | CTCCCCATCATCCCAGTCTC | CTTTGCGCTTCACTTCCACT |
| *Cla002932* | CACGGCCATTCTCTTCAAGG | GCTTGCCCACTAATACCAGC |
| *Cla002791* | CATCTTCAGCCGTCAAAGGG | TTTGAGCCACTTGACCATGC |
| *Cla015195* | GGACGCTCACTATAAGGCCT | CCCTGAATTGGCTTTGGGTC |
| *Cla002788* | CACCACCTCCAACACCTACT | GCCGGTGGTGATCAATTCTC |
| *Cla011567* | CGGTGGCCTAAAATCGAGTG | AAGACAACCAAGCCCAACAC |
| *Cla010146* | ATGACCAATTCCTCGTCCCC | TTGGCGCGATTTCTTCTCTG |
| *Cla011204* | GGGTTCGGATGCTTTACACC | GGTCGGCGATAGGGTAGAAA |
| *Cla020067* | GCTTACCATCAACGGGCATT | TGCAACTGTGAGCTGTGAAC |
| *Cla007450* | AGGTTCACTCACGATGCTGA | GGTCGTTGTTCCGGTAATGG |
| *Cla007792* (ACT*) | CCATGTATGTTGCCATCCAG | GGATAGCATGGGGTAGAGCA |

* Reference gene (ACT) for qRT-PCR analysis

**Table S7** *Arabidopsis thaliana* Ortholog genes to the selected candidate genes that substantially responded to low N (0.2 mM) compared with high N (9 mM) in the leaf and root of watermelon

| Gene ID | Functional annotation  (watermelon) | Ortholog genes | Gene name | Functional annotation | Organism | E value | Reference |
| --- | --- | --- | --- | --- | --- | --- | --- |
| *Cla010066* | Nitrate transporter | [AT5G60780](http://www.arabidopsis.org/servlets/TairObject?type=gene&id=133978) | Arabidopsis thaliana nitrate transporter 2.3, ATNRT2.3, Nitrate transporter 2.3, NRT2.3 | High affinity nitrate transporter | *Arabidopsis thaliana* | 3e-10 | [1,2] |
| *Cla009721* | Nitrate transporter | [AT1G59740](http://www.arabidopsis.org/servlets/TairObject?type=locus&name=AT1G59740) | ATNPF4.3, NPF4.3, NRT1/ PTR family 4.3 | Major facilitator superfamily protein | *Arabidopsis thaliana* | 0.024 | [3] |
| *Cla012765* | Nitrate transporter | [AT5G13400](http://www.arabidopsis.org/servlets/TairObject?type=locus&name=AT5G13400) | [PTR2 family](http://www.ebi.ac.uk/interpro/IEntry?ac=IPR018456) | Major facilitator superfamily protein | *Arabidopsis thaliana* | 2e-55 | [4] |

1. Bi YM, Wang RL, Zhu T, Rothstein SJ. Global transcription profiling reveals differential responses to chronic nitrogen stress and putative nitrogen regulatory components in Arabidopsis. BMC Genomics. 2007;8:281.

2. Deeken WR, Julia C. Engelmann, Marina Efetova, Tina Czirjak, Tobias Muller, Werner M. Kaiser, Olaf Tietz, Markus Krischke, Martin J. Mueller, Klaus Palme, Thomas Dandekar, Rainer Hedrich. An integrated view of gene expression and solute profiles of Arabidopsis tumors: A aenome-wide approach. Plant Cell. 2006;18:3617-3634.

3. Li JY, Fu YL, Pike SM, Bao J, Tian W, Zhang Y, Chen CZ, Zhang Y, Li HM, Huang J, Li LG, Schroeder JI, Gassmann W, Gong JM. The Arabidopsis nitrate transporter NRT1.8 functions in nitrate removal from the xylem sap and mediates cadmium tolerance. Plant Cell. 2010;22:1633-1646.

4. Tsay YF, Chiu CC, Tsai CB, Ho CH, Hsu PK. Nitrate transporters and peptide transporters. FEBS Lett. 2007; 581:2290-2300.

**
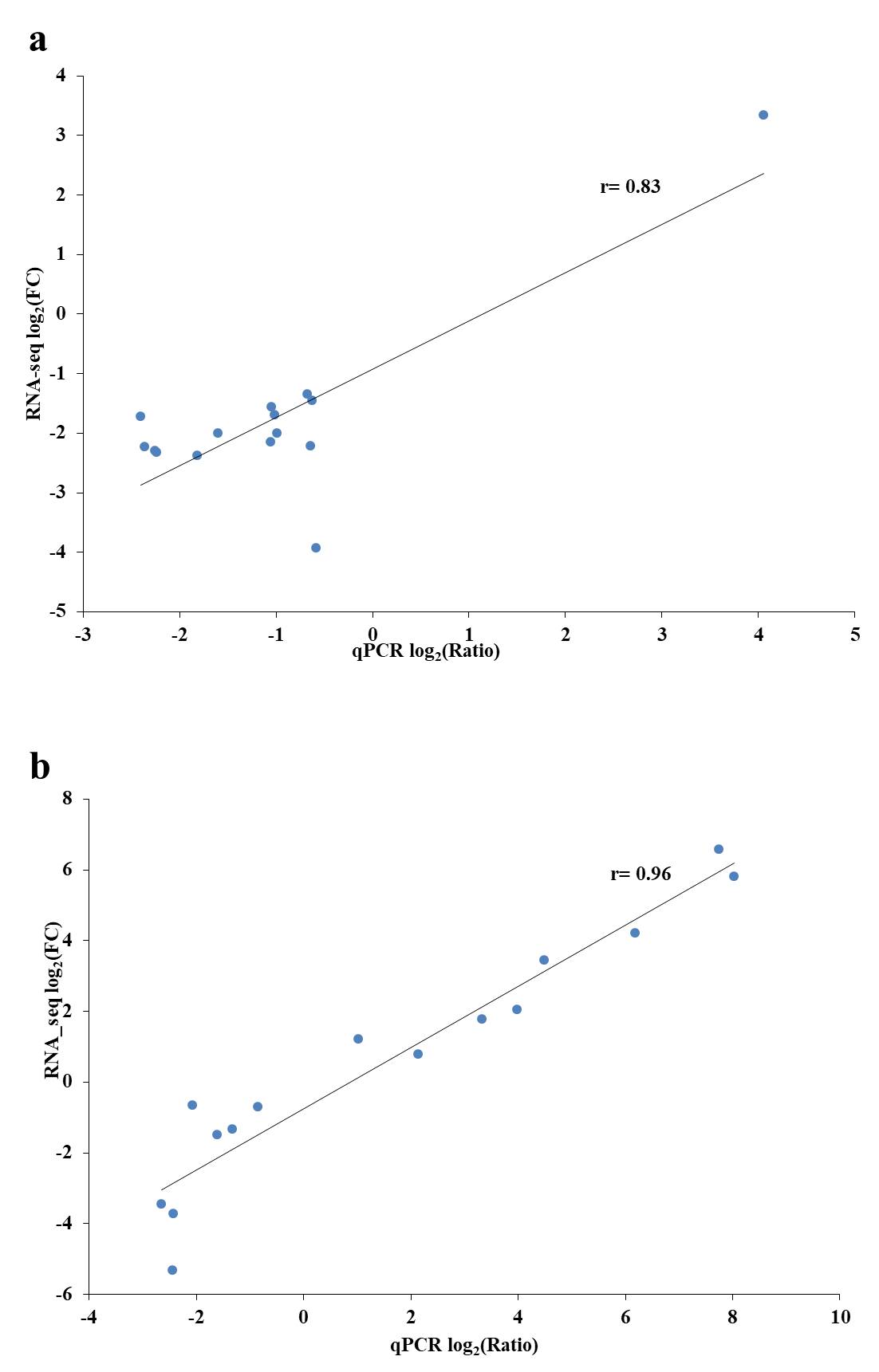
**

**Fig. S1** Correlation between expression value of selected genes obtained by RNA-seq and qPCR in the leaf (a) and root (b) tissues of watermelon seedlings grown under hydroponic conditions exposed to different levels of N (0.2 mM and 9 mM) for 14 days. FC: fold change; r: correlation coefficient

**
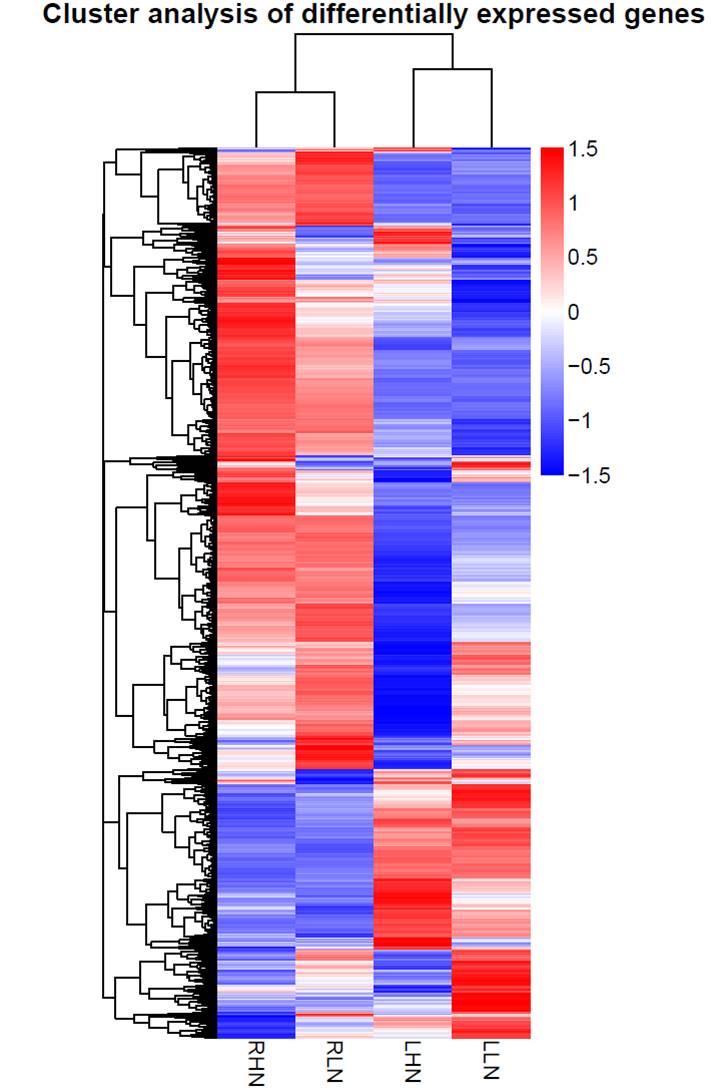
**

**Fig. S2** Hierarchical cluster analysis map presenting differential gene expression in the leaf and root of watermelon grown under hydroponic conditions at 0.2 mM and 9 mM N. LHN: leaf high N (9mM); LLN: leaf low N (0.2 mM); RHN: root high N (9mM); RLN: roots low N (0.2 mM). Samples for transcriptome analysis were harvested after 14 days of N treatment

**
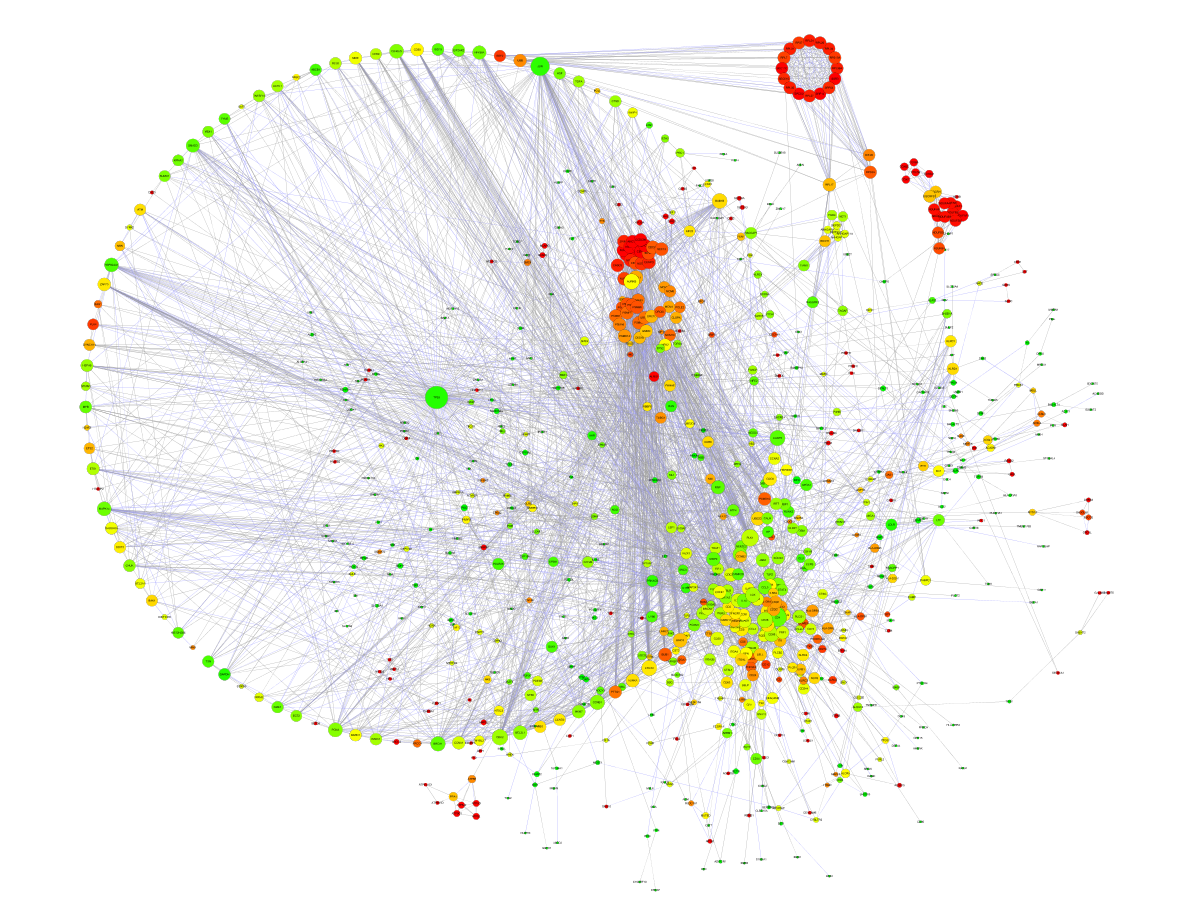
**

**Fig. S3** The cytoscape presenting protein interaction network analysis of differentially expressed genes of leaf and root of watermelon grown under hydroponic conditions at 0.2 mM and 9 mM N
